# A Focal Inactivation and Computational Study of Ventrolateral Periaqueductal Gray and Deep Mesencephalic Reticular Nucleus Involvement in Sleep State Switching and Bistability

**DOI:** 10.1523/ENEURO.0451-19.2020

**Published:** 2020-11-04

**Authors:** Kevin P. Grace, Richard L. Horner

**Affiliations:** 1 Institute of Medical Science, University of Toronto, Toronto, Ontario M5S 1A8, Canada; 2 Department of Medicine, University of Toronto, Toronto, Ontario M5S 1A8, Canada; 3 Department of Physiology, University of Toronto, Toronto, Ontario M5S 1A8, Canada; 4 Department of Neurology, Beth Israel Deaconess Medical Center and Division of Sleep Medicine, Harvard Medical School, Boston, MA 02215

**Keywords:** EEG, microdialysis, pharmacology, REM sleep, state dynamics

## Abstract

Neurons of the ventrolateral periaqueductal gray (vlPAG) and adjacent deep mesencephalic reticular nucleus (DpMe) are implicated in the control of sleep-wake state and are hypothesized components of a flip-flop circuit that maintains sleep bistability by preventing the overexpression of non-rapid eye movement (NREM)/REM sleep intermediary states (NRt). To determine the contribution of vlPAG/DpMe neurons in maintaining sleep bistability we combined computer simulations of flip-flop circuitry with focal inactivation of vlPAG/DpMe neurons by microdialysis delivery of the GABA_A_ receptor agonist muscimol in freely behaving male rats (*n* = 25) instrumented for electroencephalographic and electromyographic recording. REM sleep was enhanced by muscimol at the vlPAG/DpMe, consistent with previous studies; however, our analyses of NRt dynamics *in vivo* and those produced by flop-flop circuit simulations show that current thinking is too narrowly focused on the contribution of REM sleep-inactive populations toward vlPAG/DpMe involvement in REM sleep control. We found that much of the muscimol-mediated increase in REM sleep was more appropriately classified as NRt. This loss of sleep bistability was accompanied by fragmentation of REM sleep, as evidenced by an increased number of short REM sleep bouts. REM sleep fragmentation stemmed from an increased number and duration of NRt bouts originating in REM sleep. By contrast, NREM sleep bouts were not likewise fragmented by vlPAG/DpMe inactivation. In flip-flop circuit simulations, these changes could not be replicated through inhibition of the REM sleep-inactive population alone. Instead, combined suppression of REM sleep active and inactive vlPAG/DpMe subpopulations was required to replicate the changes in NRt dynamics.

## Significance Statement

Sleep separates into two distinct states: rapid eye movement (REM) and non-REM (NREM) sleep. The circuit mechanisms underlying this bistability have not been defined. Neurons in the ventrolateral periaqueductal gray and adjacent deep mesencephalic reticular nucleus (vlPAG/DpMe), namely those with REM sleep-inactive discharge profiles, are hypothesized to be negative-regulators of NREM-to-REM sleep transitions. We show that the main effect of vlPAG/DpMe inactivation is a loss of sleep bistability that originates mainly in REM sleep, which suggests the important added involvement of the vlPAG/DpMe REM sleep-active cell population. Simulations of flip-flop circuitry support the participation of REM sleep-active and sleep-inactive vlPAG/DpMe cell groups in a noisy, asymmetric flip-flop switch that contributes to the bistability and robustness of sleep switching.

## Introduction

Mammalian sleep separates into two distinct states: rapid eye movement (REM) sleep and non-REM (NREM) sleep. One of the fundamental characteristics of REM sleep is that depriving an animal of this state will result in a postdeprivation rebound, i.e., the propensity of future REM sleep sates depends on the occurrence of past REM states (at least over long time scales; Benington and Heller, 1994a,b; Franken, 2002). This rule of the REM sleep control system has important implications. First, it implies that some component(s) of the system must track the inter-REM sleep bout interval (i.e., the nervous system stores a history of REM sleep). Second, the switching circuitry responsible for transitioning between NREM and REM sleep must be sensitive to changes in this history. Nevertheless, sleep is normally bistable, implying that NREM-REM sleep switching circuitry is configured so as to avoid generating intermediary states. If switching circuitry responded continuously to the build-up of REM sleep drive over the inter-REM sleep interval, intermediate sleep states would dominate. Therefore, to minimize the occurrence of intermediate states, the NREM-to-REM sleep switching circuitry should contain a bistability maintenance mechanism. While many neuronal populations have been implicated in the control of NREM and REM sleep, the circuit logic underlying the bistability of sleep is poorly understood. It has been suggested that sharp transitioning between sleep-states is the product of a flip-flop circuit arrangement, a mutual inhibitory interaction between NREM-active and REM sleep-active cell populations (Lu et al., 2006a; Sapin et al., 2009).

Neuronal populations of the ventrolateral periaqueductal gray and the adjacent deep mesencephalic reticular nucleus (vlPAG/DpMe) have been suggested to participate in a flip-flop circuit for REM sleep generation (Luppi et al., 2006; Saper et al., 2010; Fraigne et al., 2015). Based on measures of c-fos expression following REM sleep deprivation and recovery, the vlPAG/DpMe region contains some of the largest populations of both REM sleep-inactive and REM sleep-active neurons in the brainstem (Sapin et al., 2009). Extracellular single-unit recording and single-cell calcium imaging studies have also profiled these opposing subpopulations (Crochet et al., 2006; Weber et al., 2018), with particular focus on GABAergic neurons.

Inactivation of vlPAG/DpMe neurons reliably increases REM sleep amounts in rats (Lu et al., 2006a; Sapin et al., 2009), mice (Hayashi, 2015; Weber et al., 2015), cats (Petitjean et al., 1975; Sastre et al., 1996; Crochet et al., 2006), and guinea pigs (Vanini et al., 2007) by means of lesioning, focal muscimol delivery, chemogenetic inhibition, and optogenetic stimulation of ventral medullary GABAergic axons in the vlPAG/DpMe. Reported increases in REM sleep time range from 130% to 380% of baseline and, in most cases, result from increases in both the duration and frequency of episodes. A smaller increase in REM sleep (∼20% increase from baseline) was reported following virally-mediated ablation of vlPAG GABAergic neurons (Weber et al., 2018). Based on these findings, current hypotheses of vlPAG/DpMe involvement in REM sleep control focus on the role of REM sleep-inactive GABAergic neurons in gating NREM-to-REM sleep transitioning (Lu et al., 2006a; Sapin et al., 2009; Saper et al., 2010). Notably, optrode recording of GABAergic vlPAG neurons has revealed that some vlPAG cells are inhibited by laser stimulation, suggesting that they receive inhibitory inputs from channelrhodopsin-expressing vlPAG GABAergic neurons. Of those cells, half were identified as having a REM sleep-active activity profile (Weber et al., 2018). This raises the possibility that inhibitory signaling between vlPAG/DpMe REM sleep-active and sleep-inactive subpopulations, potentially in the form of a mutually inhibitory flip-flop switch, are important for the control of sleep state switching.

However, there is little direct evidence that neurons within the vlPAG/DpMe participate in a bistability mechanism. In order to measure changes in sleep stability, NREM-REM sleep intermediate states (NRt) should be quantified. Such states develop naturally during normal transitioning from NREM-to-REM sleep (Benington et al., 1994; Boucetta et al., 2014). Here, we compare changes in NRt dynamics resulting from inactivating vlPAG/DpMe neurons *in vivo* to those resulting from simulated inactivations of flip-flop switching circuitry. Our data are not consistent with the inhibition of a REM sleep-inactive population per se. *In vivo*, we observed increased amounts of NRt, which primarily fragmented REM sleep. This and other findings were best accounted for by simulated inhibition of both REM sleep-active and REM sleep-inactive cell groups participating in a noisy, asymmetric flip-flop switch that contributes to the bistabilty and robustness of sleep switching.

## Materials and Methods

### Animal care

All animal experimentation is in accordance with the Society for Neuroscience’s Policies on the Use of Animals in Neuroscience Research. All animal procedures were performed in accordance with the University of Toronto’s animal care committee’s regulations. Experiments were performed on a total of 25 male Wistar rats [Charles River; mean body weight = 285.2 ± 1.6 g (±SEM), range 276–300 g]. Rats were housed in groups before surgery. Rats were maintained on a 12/12 h light/dark cycle [lights on at 7 A.M. (Zeitgeber Time (ZT) 0)], and had free access to food and water.

### Surgery

Rats were implanted with electrodes for chronic recording sleep-wake state: electroencephalogram (EEG) and electromyogram (EMG) of the trapezius muscle. Sterile surgery was performed under general anesthesia induced with isoflurane (3.5%). Rats were intraperitoneally injected with buprenorphine (0.03 mg kg^−1^) to minimize postoperative pain, atropine sulfate (1 mg kg^−1^) to minimize airway secretions, and saline (3 ml, 0.9%) for fluid loading. A surgical plane of anesthesia, as judged by abolition of the pedal withdrawal and corneal blink reflexes, was maintained with isoflurane (2–2.5%) administered with an anesthesia mask placed over the snout.

Microdialysis guides (CXG-6; Eicom) were targeted to a position 0.8 mm anterior to lambda (as defined by Paxinos and Watson (1998), as the midpoint of the curve of best fit along the lambdoidal suture), 0.6 mm lateral to the midline, and 3.7 mm ventral to lambda. These coordinates correspond to a position 2.5 mm above the targeted region of drug delivery in the vlPAG/DpMe (Paxinos and Watson, 1998). Dummy cannulas were placed inside the microdialysis guides to keep them free of debris until the day of the experiment. Microdialysis probes projected an additional 2.5 mm from the guide and so targeted the vlPAG/DpMe. Following surgery, rats were housed individually in separate cages while maintaining visual, olfactory, and auditory contact with other rats. Rats recovered for at least 6 d before experiments were conducted. Sleep time, particularly REM sleep, is sensitive to housing conditions (Febinger et al., 2014). REM sleep bouts in rodents are more fragmented when animals are housed in groups. The sleep recordings performed in this study require tethering and individual housing. Housing rats individually in the postoperative period was intended to promote stable sleep conditions in the days leading up to the sleep recordings and to allow for accommodation to the social isolation.

### Microdialysis perfusion

The evening before the first study day, rats were placed in the recording environment and connected to a lightweight counterbalanced recording cable for habituation purposes. The recording environment consisted of a large open-topped bowl (Rodent Bowl, MD-1514, BAS Inc) mounted on a turntable (Rat Turn, MD-1404, BAS Inc), housed within an electrically-shielded and soundproofed cubicle (EPC-010, BRS/LVE Inc.). A video camera located within the cubicle allowed for continuous visual monitoring without disturbing the animal. At the time of recording cable attachment, the internal cannula was removed from the guide, and the microdialysis probe was inserted (CX-I-8-005; 19-nl tip volume; 220 μm in diameter, 500-μm-long membrane; 50,000-Da cutoff; Eicom). The probes were connected to FEP Teflon tubing (inside diameter, 0.12 mm) with this tubing connected to 1.0 ml syringes via a zero dead space switch (Uniswitch, BAS). The probes were perfused with freshly-made artificial CSF (ACSF) at a flow rate of 2.1 μl/min. It is important to note that, when using microdialysis for drug delivery, drug is introduced into the extracellular space by passive diffusion across the semi-permeable membrane of the probe. This is in contrast to pressure microinjection, which forces a volume of drug solution into the extracellular space and the initial spread of the drug occurs by bulk flow. In the case of microdialysis, drug administration does not entail any physical distortion of brain tissue in the target region, as there is no fluid volume ejected. Moreover, it is also important to keep in mind that with microinjection, drug concentration peaks at the target site immediately after the injection and then rapidly decays, whereas with microdialysis, drug suffuses and accumulates within the target region more slowly and asymptotically approaches a concentration plateau that is ∼20% of the drug concentration internal to the probe (Grace et al., 2014a). This plateau arises because of an equilibrium between the drug diffusion gradient and the tortuosity of the diffusion volume (i.e., the average impediment to free diffusion because of the physical complexity of the extracellular space)

### Experimental design

The study consisted of separate time-control (*n* = 13) and drug groups (*n* = 12). Experiments took place between ZT3 and ZT7.5 and signals were recorded continuously during this time. Between ZT3 and ZT5, ACSF was microperfused in all animals. Between ZT5 and ZT7.5, ACSF microperfusion was maintained in the time-control group, while the drug-group received microperfusion of the GABA_A_ receptor agonist muscimol (Tocris Bioscience; 85 μm in ACSF; microdialyzed solution). A previous vlPAG/DpMe inactivation study, performed in cats, used muscimol concentrations of 100, 500, and 1 mm, administered by reverse microdialysis (Eicom, probe model: A-L-50-01; 2-h microperfusion; Crochet et al., 2006). In that study, muscimol inactivation of the vlPAG/DpMe had a REM sleep enhancing effect stemming from an increase in the duration and frequency of REM sleep episodes. In the present study, data collected between ZT5 and ZT5.5 were excluded from analysis to allow microperfused muscimol to suffuse the vlPAG/DpMe region and approach its plateau concentration.

Following completion of the experiments, the rats were killed and their brains were removed to determine the position of the microperfusion sites. The locations of these sites were recorded on standard coronal drawings from the stereotaxic atlas of the rat brain prepared by Paxinos and Watson (1998). We estimated the approximate tissue volumes where muscimol-mediated suppression of neuronal activity is likely to have occurred in our animals. To generate these estimates, we used computer simulations (MATLAB; R2017a) of muscimol diffusion based on available muscimol pharmacology and diffusion data *in vivo* (further details are provided in Results, Estimating the spread of muscimol using microdialysis delivery). Simulations were run using custom MATLAB code. This code is freely available online at https://github.com/KPGrace/Grace_Horner_Eneuro2020. The code is also available as Extended Data 1.

10.1523/ENEURO.0451-19.2020.ed1 Extended Data Code accessibility statement. The included computer code is in four parts. First, is a MATLAB script entitled “flip_flop_circuit_simulation_initializer.” This code was used to initialize simulations run with SimLIFnet (available for download at https://www.mathworks.com/matlabcentral/fileexchange/50339; copyright 2015, Zachary Danziger, all rights reserved) using the simulation parameters listed in Extended Data Table 8-1. Second is a MATLAB function entitled “forceramp,” which is required by “flip_flop_circuit_simulation_initializer” and determines the profile of the R-state promoting drive. Third, is a MATLAB script entitled “intersection_finder,” which was used to identifying all points in NREM/REM state space that bound trajectory intersections occurring within 1-min-wide windows. This procedure is needed to demarcate NREM, REM, and NRt regions of state space. Fourth, is a MATLAB script entitled “drug_diffusion_simulations,” which was used to estimate the 3-dimenional spread of drug from a point source in a microinjection versus a reverse-microdialysis scenario. This code is freely available online at https://github.com/KPGrace/Grace_Horner_Eneuro2020. Download Extended Data, ZIP file.

### Signal analyses

Electrical signals were amplified and filtered (Super-Z head-stage amplifiers and BMA-400 amplifiers/filters, CWE). The EEG was filtered between 1 and 100 Hz, whereas the neck EMG was filtered between 100 and 1000 Hz. All signals were digitized at 2000 Hz (Spike 2 software, 1401 interface, CED).

The data were analyzed in consecutive 5-s time bins. The EEG signal was subjected to a fast-Fourier transform for each 5-s time bin. The power in frequency bands (2 Hz in width) spanning the 1- to 33-Hz range was calculated.

Sleep-wake states were initially identified by visual inspection, and classified into wakefulness, NREM, and REM sleep according to standard criteria. Subsequent to visual scoring we used the method described in the following section, NREM-to-REM sleep transition dynamics, to perform additional scoring of NREM/REM intermediary sleep (NRt) epochs.

### NREM-to-REM sleep transition dynamics

The algorithm for defining NRt dynamics is depicted in Extended Data Figure 2-1. The procedure used here has been described elsewhere (Grace et al., 2014b). First, the EEG was subjected to state-space analysis (Gervasoni et al., 2004; Diniz Behn et al., 2010). The objective of a state-space approach is to create a two-dimensional plot bounded by electroencephalographic variables that can be used to distinguish sleep-wake states from one another, in which the position of a single point represents the electroencephalographic state of the forebrain during a single recording epoch. (Extended Data Fig. 2-1*A–C*). The log_10_ transform of spectral power in the 1- to 19-Hz frequency range and the log_10_ transform of the ratio of spectral power in the 7- to 9-Hz and the 1- to 9-Hz frequency ranges were used to create state-space plots. Data were smoothed before plotting using a 5-epoch wide moving average (MATLAB; function: smooth). Extended Data Figure 2-1*B* shows example data from NREM and REM sleep. Following this procedure and plotting the resulting data yields two visible clusters of points, one for each sleep state. However, to analyze transitions between NREM and REM sleep states, standardized boundaries of the NREM and REM sleep state-space clusters need to be defined.

**Figure 1.**
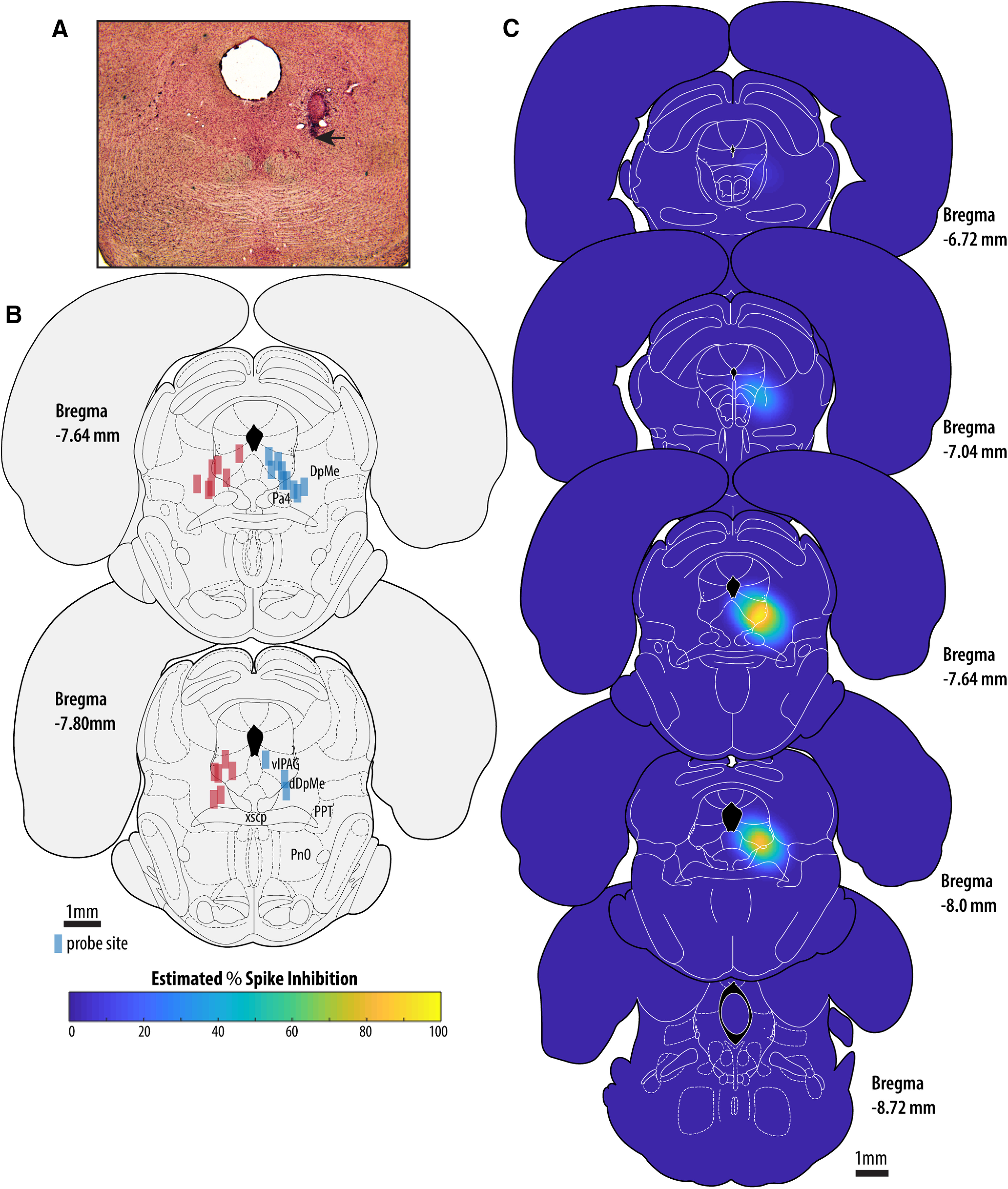
Location of microperfusion sites and prediction of the anatomic extent of muscimol-mediated inhibition. ***A***, Example site of microperfusion located at the boundary of the vlPAG and the DpMe. The black arrow indicates the most ventral point of the lesion left by the microdialysis probe. ***B***, Probe locations sites for all 25 rats (blue rectangles = muscimol group; red rectangles = control group) located between the anterioposterior level defined by the caudal pole of the paratrochlear nucleus to the caudal extent of the superior cerebellar peduncle decussation. All probes were implanted on the right side; probes positions are shown on both sides for clarity purposes. ***C***, Results of our simulation-based estimate of average PSI across all experiments in stereotaxic coordinate space.

**Figure 2.**
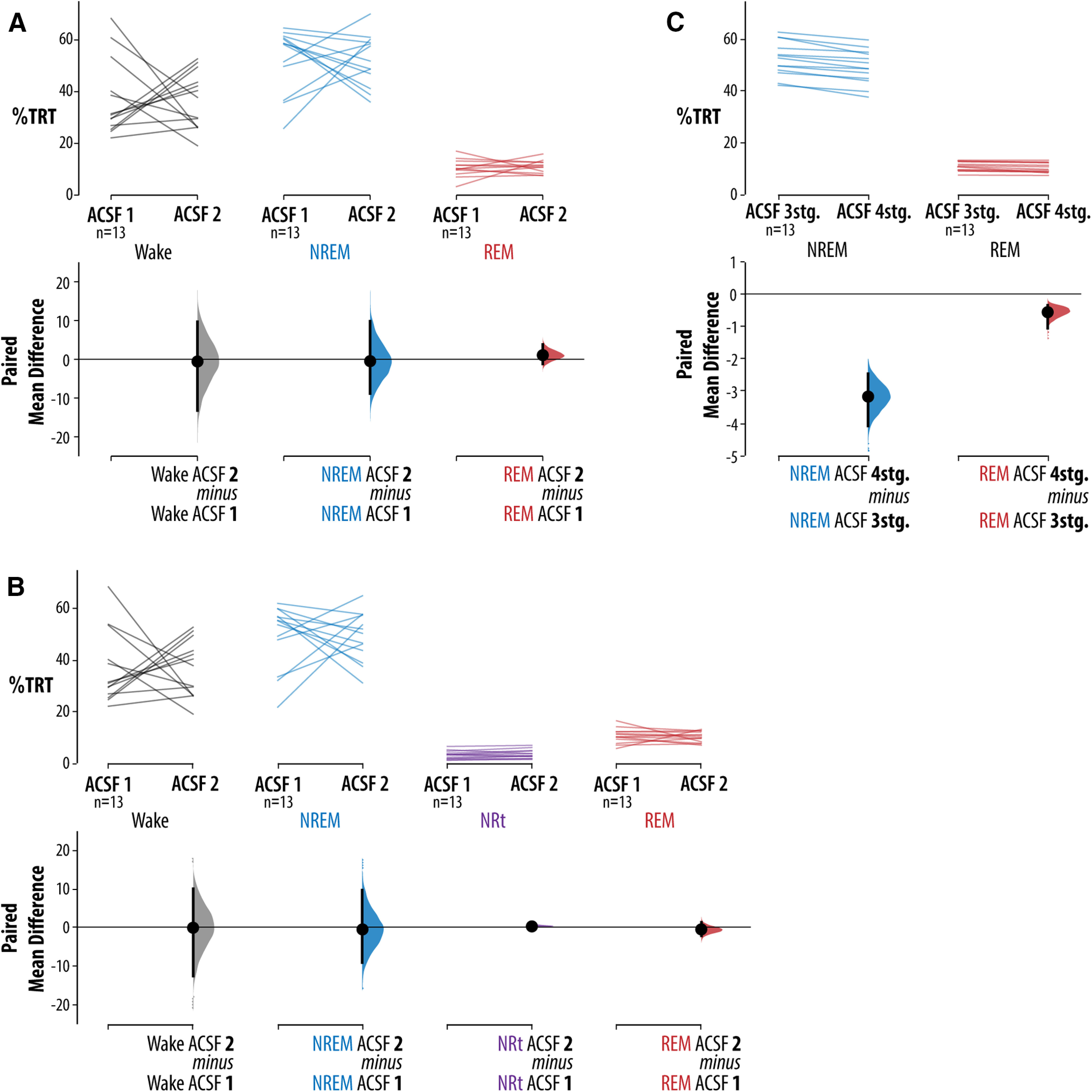
Control group validation of the NRt scoring method. ***A***, Compared levels of wake, NREM, and REM sleep between periods 1 (ZT3 and ZT5) and 2 (ZT5.5 and ZT7.5) in the control group (*n* = 13). ***B***, Same data as in ***A***, except the NRt scoring method is used. ***C***, Same data as in ***B***, except levels of sleep are expresses as a percentage of TST rather than TRT. For each comparison, the paired mean differences are shown in Cumming estimation plots. The raw data are plotted on the upper axes; each paired set of observations is connected by a line. On the lower axes, each paired mean difference is plotted as a bootstrap sampling distribution. Mean differences are depicted as dots; 95% CIs are indicated by the ends of the vertical error bars. The algorithm for defining NREM-to-REM sleep transition dynamics is illustrated in Extended Data Figure 2-1.

10.1523/ENEURO.0451-19.2020.f2-1 Extended Data Figure 2-1 Procedure for defining NREM-to-REM sleep transition dynamics. ***A***, Flow chart outlining the EEG preprocessing steps required to construct NREM/REM state-space plots. ***B***, ***C***, Example state-space plots showing clusters of points corresponding to REM and NREM sleep. ***D***, ***E***, Procedure for defining the boundaries of the REM and NREM clusters that separate them from the intervening NRt space. This procedure identifies the position of trajectory intersections within 5-epoch spans. Example trajectory intersections are shown in ***D***. All data points that bound all such trajectory intersections are fitted with a convex envelope to form the REM sleep and NREM sleep boundaries shown in ***E***. ***F***, ***G***, Example trajectories of complete NREM-to-REM transitions (***F***) and the trajectories of failed transitions through NRt space (***G***). Download Figure 2-1, TIF file.

The method used to define state boundaries is a product of our basic assumptions regarding the mechanisms underlying sleep state generation. The occurrence of distinct states of NREM and REM sleep is the result of distinct generating circuits imposing distinct boundary conditions on brain state over time (Gervasoni et al., 2004). State dynamics observed across transitions between NREM and REM sleep are assumed to be a consequence of competition between NREM and REM sleep generating circuits (i.e., a period without a dominant set of boundary conditions). Based on these assumptions, it is expected that NREM and REM sleep regions of state space, in contrast to transitionary space, contain bounded trajectories (Diniz Behn et al., 2010). Where a trajectory is constrained by a boundary, it is expected that the trajectory undergoes reversals in direction resulting in trajectory intersections. Where a trajectory is unbounded and has a directional preference, intersections should occur less often (at least over short time scales; Diniz Behn et al., 2010). These trajectory characteristics are evident in Extended Data Figure 2-1*C*,*D*, which show that state trajectories in the intervening space between NREM and REM sleep clusters exhibit a directionality or a dependency on past trajectory (i.e., second or higher-order dependence) that is seemingly absent within the clusters (i.e., first or zeroth order dependence). For each 2-h ACSF control period, we calculated the position of trajectory intersections, if any, occurring within a 5-epoch wide moving window using custom MATLAB code. This code is freely available online at https://github.com/KPGrace/Grace_Horner_Eneuro2020. The code is available as Extended Data 1. Each intersection is, by definition, bounded by four epoch points. Examples of such trajectory intersections and the surrounding points are depicted in Extended Data Figure 2-1*D*. For the example data in Extended Data Figure 2-1, all such points associated with all such trajectory intersections are plotted in Extended Data Figure 2-1*E*. Notice that following this procedure, points in the intervening space between the NREM and REM clusters are largely absent, confirming the suggestion that, in this region of state space, the trajectories have some directional preference and avoid looping. To define the boundaries of the NREM and REM sleep state space, convex envelopes were fit to each cluster (MATLAB function: convhull). To avoid cluster boundaries being disproportionately affected by extreme points, the distance of each point from the cluster centroid was calculated and points with distances greater than five standard deviations of the mean centroid distance were excluded. Extended Data Figure 2-1*E* shows examples of convex envelopes fitted to NREM and REM sleep coordinate points.

After using this procedure to separate state space into NREM, REM and transitionary regions, the onset and offset of NRt bouts can be determined. However, not all data points located in transitionary space were classified as belonging to an NRt bout. We adopted specific scoring rules to prevent our analysis being overly influenced by transient boundary crossings. To score epochs as transitionary sleep we used the following criteria, adapted from the American Sleep Disorders Association’s EEG scoring rules for EEG arousals from sleep (American Sleep Disorders Association, 1992): (1) NRts must be preceded by two consecutive epochs of NREM or REM sleep (i.e., two consecutive points located within a sleep state boundary); (2) following NRts, two consecutive epochs located within a sleep state boundary are required to score NREM-to-REM sleep or REM-to-NREM sleep transitions; (3) where an excursion into transitionary space is terminated by an arousal or by reentry into the state of origin, the excursion into transitionary space must be at least two epochs in duration; (4) bouts of NRt interrupted by visually scored arousals, lasting three epochs or less, are not considered separate bouts; (5) for excursions from NREM state space into transitionary space, only trajectories traversing transitionary space from right-to-left (i.e., toward REM sleep state space) were scored as NRt; and (6) excursions into transitionary space are scored as transitionary epochs only when such trajectories pass through the region of transitional space demarcated by the set of NREM-to-REM sleep transition trajectories. This last scoring rule is depicted in Extended Data Figure 2-1*F*,*G*. Extended Data Figure 2-1*F* shows the trajectory, through NRt space, of multiple complete transitions between NREM and REM sleep. These trajectories demarcate the NRt space depicted in Extended Data Figure 2-1*G*, which is in turn used to define valid NRts that are not complete transitions between NREM and REM sleep. Therefore, NRt space is defined by the spectral characteristics of complete transitions between NREM and REM sleep, i.e., NRt bouts, whether they originate in NREM or REM sleep, are, by definition, consistent with the spectral characteristics of bona fide transitionary sleep. The result of this complete procedure is an edited/standardized version of the original manual sleep scoring that additionally includes bouts of NRt (Extended Data Fig. 2-1*H*).

### Design overview of the flip-flop circuit computer simulations

This section provides a general overview of the design of, and the rationale supporting, our flip-flop circuit simulations. The proceeding sections provide the technical details of the simulations.

Reliably inferring higher order circuit logic from experimentally induced changes to sleep-state dynamics is often not possible without a computational approach, because the ways in which state dynamics are shaped and constrained by circuit configuration are often not intuitive. Consistent with prevailing hypotheses and previous modeling studies (Kumar et al., 2012; Rempe et al., 2010; Dunmyre et al., 2014), we assume that the switching elements of the REM sleep control circuitry receive a slowly varying, graded REM sleep drive signal. We assume that bistability (i.e., the relative absence of NRt) is created by interaction between NREM sleep promoting and REM sleep promoting neuronal pools. Previous modeling studies have aimed to account for the observed statistical properties of sleep-wake state transitioning, over many sleep-wake cycles, using systems of NREM and REM sleep homeostatic drives interacting with flip-flop circuits (Kumar et al., 2012; Rempe et al., 2010; Dunmyre et al., 2014). These models do not strictly consider intermediary states, which are of particular relevance to biological flip-flops. The aim of our circuit simulations is to evaluate whether the effects, on NRt dynamics in particular, of vlPAG/DpMe inhibition are consistent with the predicted changes in intermediary state dynamics resulting from inhibition of one or both pools of a simple flip-flop circuit.

Here, we use simulations of a simple system including a flip-flop circuit with input from a monotonically increasing linear drive signal. Unlike previous modeling work, we purposefully do not include a negative feedback in the circuits to model homeostatic modulation of the REM promoting drive. Homeostasis and bistability, while related, are nevertheless separable features of the REM sleep control circuitry. Our simulations focus on sleep bistability, i.e., NRt dynamics and bout statistics within bouts of NREM and REM sleep. The flip-flop circuits that we simulate are composed of two pools of leaky integrate-and-fire (LIF) type neurons that mutually inhibit each other with random connectivity. The output states of the switch are determined by comparing average population spiking activity. We assume that naturally occurring NRt EEG states are a reflection of intermediary firing rates in the underlying NREM and REM sleep promoting populations. Therefore, intermediate states produced by circuit simulations are likewise defined as periods of intermediate firing in the opposing populations of the flip-flop switch. Importantly, in these simulations, the occurrence of intermediary states is not guaranteed and is instead dependent on particular simulation features that are biologically relevant. NRt occurrence requires inclusion of sources of stochastic variation, random connectivity between the flip-flop pools, and variation in the synaptic weighting between the flip-flop pools. Lastly, to reflect the inherent asymmetry of sleep switching (i.e., NREM sleep often gates REM sleep and is more abundant), we made the inhibitory output of the NREM representative pool stronger than the REM representative pool. To summarize, we simulated a ramping input signal into a flip-flop switch composed of two pools of noisy, tonically active LIF-type neurons that are mutually inhibitory, randomly connected, and asymmetrically weighted. The technical details of the circuit simulations are described in the following section.

### Technical details of flip-flop circuit computer simulations

Simulations were run using MATALB (SimLIFnet function). This code is freely available online at https://www.mathworks.com/matlabcentral/fileexchange/50339 (copyright 2015, Zachary Danziger, all rights reserved). Simulations were run using a Dell Optiplex 7050 with an Intel Core i7-7700 processor, 16GB RAM, and 64-bit Windows 10 operating system (total simulation time ∼27 h). All model parameters are listed in Extended Data Table 8-1. We have made the code for initializing “SimLIFnet” simulations with the parameters/settings in Extended Data Table 8-1 and for running the experimental manipulations freely available. This code is freely available online at https://github.com/KPGrace/Grace_Horner_Eneuro2020. The code is available as Extended Data 1. Simulated networks were composed of LIF type neurons (Stein, 1965; Knight, 1972; Burkitt, 2006; Brunel and van Rossum, 2007). The method of numerical integration is a fixed step size Euler method. As with all integrate-and-fire neuronal models, spikes are not explicitly modelled but rather when membrane potential reaches a set threshold a spike is registered and membrane potential immediately resets to the resting potential. All parameters have been non-dimensionalized and scaled such that the spiking threshold is one and resting potential is zero and time is rescaled by the ratio of the membrane time constant to the conductance (Lewis and Rinzel, 2003). The derivative of the membrane potential is given by Lewis and Rinzel (2003; their Eq. 5):

$$\frac{dvi}{dt}=-vi+I_{applied}+Wji\overset{0}{\underset{n=-\infty }{\sum }}s_{ij}(t-t_{n,j}),$$

where:

$$s_{ij}(t-t_{n,i})=\alpha^{2}(t-t_{n,i})e^{-\alpha \left(t-t_{n,i}\right)}.$$

The expression describes the dynamics of current input to neuron *i* based on the time history of spikes from neuron *j*. Where *t_n,i_* represents the past spike times from neuron *j*, *t* is the current time, *a* is a constant representing the speed of the synapse, W*ji* is the weight of the connection from the *j*-th neuron to the *i*-th neuron, and I*_applied_* consists of the noisy excitatory bias current and the ramping input signal.

Flip-flop circuits are composed of two mutually inhibitory pools of 25 neurons (i.e., pools N and R, meant to represent NREM and REM active pools, respectively). We randomly generated 60 connectivity matrices that varied the connectivity and synaptic weighting between neurons in the N-pool and R-pool. For each connectivity matrix, N_j_→R_i_ and R_j_→N_i_ connection probability was 0.5. All connection weights were negative (i.e., inhibitory). All N_j_→N_i_ and R_j_→R_i_ connection weights were set to zero (i.e., auto-inhibition within N-pool and R-pool was removed). All neurons in the N-pool and R-pool received a number of “applied currents.” Each N and R neuron received: (1) a positive bias current (and therefore tended to produce tonic firing patterns) and (2) a zero-mean Gaussian noise current (amplitude = 1.5). Depending on the simulation, either the N-pool or the R-pool received the ramping input from a pool of input neurons, which will cause the switch to change state from “N-high/R-low” to “R-high/N-low.” The ramping current received by the input neurons was held at zero for the first 2000 simulation iterations and increased linearly at a rate of +0.06/iteration for the remaining 6000 iterations. The connection probability from input neurons to N and R neurons was 1.0 and the connection weight remained constant. We simulated two ramping input scenarios. In the first scenario, the ramping R-state drive input produces excitation of the R group of neurons via excitatory input neurons and in the second scenario the ramping input inhibits the N group of neurons via inhibitory input neurons. The outputs of the simulation were taken as the average spike rates for the N-pool and R-pool, taken each 20 iterations (i.e., yielding 400 total epochs). These data were smoothed using the MATLAB function: smooth (smoothing window = 3 epochs). Following smoothing, the difference in population firing rate was calculated for each epoch (R-pool minus N-pool firing). Using this difference over time, we scored the output of each network into discrete states: N, R, and NRt. The firing rate difference thresholds for the scoring of N, R, and NRt states were calculated from baseline simulations and are included in Extended Data Table 8-1. Differences in input to the N-pool and R-pool, in experiment sets 1–4 versus sets 5–8 led to different dynamic ranges of spike rate. To normalize the baseline switching behavior between experiment sets 1–4 versus sets 5–8, we applied separate firing rate difference thresholds when scoring state transitions. Switches were considered to be in the N-state for epochs where the mean R-minus-N firing difference fell below the N-state threshold and in the R-state for epochs where the R-minus-N firing difference was above the R-state threshold. All other epochs were scored as NRt. Transitions between states were only scored where state changes persisted for at least two consecutive epochs.

### Setting flip-flop synaptic weights and excitatory bias currents

The behavior of the flip-flop circuits used in these simulations is very sensitive to the weighting of the synaptic connections between N-pool and R-pool and the level of excitatory bias current applied to the neurons. Before simulating inhibition of N-pool and/or R-pool, we set the baseline behavior of the flip-flop switch by appropriately tuning the synaptic weights and applied bias currents. For these simulations, the ramping R-state drive was omitted. Nevertheless, spontaneous switching occurred because of the effect of current noise. The steps that were followed to select these parameter values are depicted in Extended Data Figure 8–2. Connection weighting was tuned before setting the level of excitatory bias current. Connection weights were initially set between (0) and (−1). Initial connection weights were divided by a factor, *d*, ranging from 1.8 to 2.8 in 0.1-unit increments. N_j_→R_i_ weights were changed independent of R_j_→N_i_ weights. This makes for 121 combinations of N_j_→R_i_ of and R_j_→N_i_ weighting (Extended Data Fig. 8-2*A*). To control for the variability in switch behavior introduced by the applied noise currents and connectivity randomization, we performed 25 simulations per weighting combination (3025 total weighting simulations). Initial membrane voltages for N-pool neurons were set such that all simulations started in an N-state. For each weighting combination, we calculated the difference in population firing rate over time for each of the 25 simulations as described in the preceding section. From these data, we created histograms showing the frequencies of binned R-minus-N firing rate differences. Examples of such histograms for particular weighting combinations are shown in Extended Data Figure 8-2*B–D*. Bimodal histograms indicate flip-flops that spontaneously switch between N-state and R-state. Where the distributions become more unimodal, the parameter values are causing the switches to: (1) become trapped in either an N-state or R-state (Extended Data Fig. 8-2*D*), or (2) lack bistability and mainly adopt intermediate firing rates (e.g., parameter combinations in the bottom right corner of Extended Data Fig. 8-2*E*). We chose a combination of R_j_→Ni and N_j_→R_i_ connection weights where N_j_→R_i_ weights are slightly stronger (N_j_→R_i_ synaptic weight range = 0 to −0.48; for *d *=* *2.1) than R_j_→Ni synaptic weights (range = 0 to −0.4; for *d *=* *2.5). This results in dominance of the N-state while maintaining the tendency of the circuit to periodically transition into NRt and the R-state. After setting the flip-flop synaptic weights, we followed a similar procedure to set the excitatory bias currents for the N-pool and R-pool neurons. Bias currents were varied from 1.5 to 2.5 in 0.1-unit increments. This resulted in a simulation space with 121 bias current combinations, which were simulated 25 times each. We chose to set the initial bias currents to 2.0 for both the N-pool and R-pool (Extended Data Fig. 8-2*F*). This combination preserved the switching behavior selected for in the preceding weighting simulations.

### Design of flip-flop experiment simulations

We simulated three experimental conditions. To simulate neuronal inactivation (i.e., increased inhibitory current) we reduced the excitatory bias current in select pools. These changes in the applied current are included in Extended Data Table 8-1. We simulated inactivation of the N-pool, the R-pool, and the combination of N-pool and R-pool for each randomly generated circuit configuration. For some connectivity matrices, the R-state dominated from the onset of simulations despite the dominance of the N_j_→R_i_ inhibitory connection weights (13/60 networks: these connectivity matrices were excluded). Therefore, the total number of simulations was 376: two input scenarios (ramp into R-pool and ramp into N-pool) × four experimental conditions (baseline, N inactivation, R inactivation, N and R inactivation) × 47 randomized flip-flop connectivity matrices. The objective of these simulations was to define characteristic changes in flip-flop switch output, in terms of the bouts statistics of N, R, and particularly NRt, that occur in response to multiple possible flip-flop inactivation experiments

### Code accessibility

The code created for this paper is freely available online at https://github.com/KPGrace/Grace_Horner_Eneuro2020 and is available as Extended Data 1.

### Statistical analysis

Data were analyzed using estimation statistics (Cumming, 2014). The statistical analyses and data plotting were performed using a MATLAB-based analysis package developed by Ho and colleagues (DABEST version 1.0.0.0; Ho et al., 2019). This code is freely available online at https://www.mathworks.com/matlabcentral/fileexchange/65260-dabest-matlab (copyright 2019, Tayfun Tumkaya, all rights reserved). Effect sizes were reported as paired mean differences together with 95% confidence intervals (CIs). Bootstrapping was used to compute the mean difference sampling distributions. For bootstrapping, 5000 samples were taken. CIs were bias corrected and accelerated. When comparing effects from our *in vivo* dataset with those from simulated experiments, we standardized effect sizes using Cohen’s *d* (i.e., standardized mean difference).

## Results

### Estimating the spread of muscimol using microdialysis delivery

Intuitions regarding the degree of spread of focally applied drugs in the brain can vary widely. Therefore, we suggest that it is instructive to provide an estimate of the approximate tissue volumes where muscimol-mediated suppression of neuronal activity is likely to have occurred in our animals. To generate these estimates, we used computer simulations of muscimol diffusion based on available muscimol pharmacology and diffusion data *in vivo*. A previous study from Arikan et al. (2002) measured changes in spontaneous neural activity surrounding microinjections of titrated muscimol before quantifying tissue levels of bound muscimol using autoradiography. Over a 2.5-h period after injection, the maximum tissue volumes where muscimol produced any observable suppression had a radius of 2 mm. Autoradiography indicated that the concentration of muscimol at the fringe of this volume was ∼10 nm. This is consistent with a study by Rijal and Gross (2008), which calculated the dose–response relationship between muscimol concentration and percent changes in spontaneous spiking. That study estimated that the effect of muscimol on spontaneous spiking approaches zero at concentrations near 10 nm. From this study we can compute the expected percent spike inhibition (PSI) given estimates of nanomolar muscimol concentration ([mus]):

$$PSI=4e^{-7}{\left[mus\right]}^{3}-0.0007{\left[mus\right]}^{2}+0.45[mus].$$

We simulated diffusion in both microinjection and microdialysis scenarios to relate the microinjection findings of Arikan et al. (2002) to our own microdialysis experiments. For important considerations regarding drug concentration profiles over time when using microinjection versus microdialysis administration, see Materials and Methods, Microdialysis perfusion. Neuronal inhibition volumes reported by Arikan et al. (2002) were obtained using 1-μl microinjections containing 20-μg muscimol administered over 30 min, in contrast to the present study, where we used reverse microdialysis delivery of a 85 μm solution. Here, we conservatively ignore the extracellular bulk flow induced by pressure microinjection and the difference in microinjection initial volume (1 μl) as compared with our microdialysis initial volume (19 nl, the volume of the microdialysis probe tip). For the purposes of simulation, we set the source volume to be equal (19 nl) for both simulations. Therefore, a 30-min microinjection of 20-μg muscimol (we treated each iteration of the simulation as a 1-min diffusion time) yields 0.667 μg/iteration, which is equal to 307 mm/iteration given a 19-nl source volume. Simulations were conducted by applying the Smooth3 function to a 250 × 250 × 250 matrix in MATLAB (convolution kernel size = 3; Gaussian filter with a SD of 1.2), where a single central cell was treated as the source volume. For the simulation of microinjection, the initial source volume concentration of 307 mm was reset for the first 30 iterations, whereas for microdialysis simulations the initial source volume concentration of 0.085 mm was reset for all 150 iterations. The standard deviation of the Gaussian filter was set such that the concentration at the matrix coordinate adjacent to the source volume plateaued at 20% of the source volume concentration (in the case of the microdialysis simulation). This value is taken from measurements in agarose tissue phantoms of permanganate concentrations adjacent to a microdialysis diffusion source [molecular weight (MW) permanganate =119 g/mol vs MW muscimol = 114 g/mol; Grace et al., 2014a]. After converting estimated muscimol concentration to PSI, we chose the diffusion distance where values dropped below 1% spike inhibition (equal to ∼2.5 nm muscimol) in our matrix to correspond to the boundary of the 4-mm-wide volume where Arikan et al. (2002) measured changes in spontaneous neural activity surrounding muscimol microinjections. The diffusion distance where concentration dropped below 1% spike inhibition in the case of microdialysis simulation was 61% of that for microinjection simulation after 150 smoothing iterations. Using this distance and the positions of the microperfusion sites in our experiments we were able to produce an estimate of average PSI across all experiments in stereotaxic coordinate space (Fig. 1).

Figure 1 shows the location of the micro-perfusion sites from both experimental groups. Figure 1*A* shows an example microperfusion lesion located at the boundary of the vlPAG and the dorsal aspect of the DpMe. Figure 1*B* shows that for all 25 rats, probe locations were located in the combined vlPAG/DpMe region, from the anterioposterior level defined by the caudal pole of the paratrochlear nucleus to the caudal extent of the superior cerebellar peduncle decussation. Figure 1*C* shows the results of our simulation-based estimate of the approximate tissue volumes where muscimol-mediated suppression of neuronal activity is likely to have occurred in our animals over a 2.5-h period. Our simulations indicate that spike inhibition is expected to be concentrated in the vlPAG and the DpMe. Importantly, we do not expect high levels of spike inhibition at important sites for REM sleep regulation located caudally to the vlPAG and the DpMe, i.e., pre-locus coeruleus, the laterodorsal and sublaterodorsal tegmental regions.

### Evidence supporting the reliability of the NRt scoring method

To test the involvement of vlPAG/DpMe neurons in the control of sleep bistability we quantified bistability in the form of NRt. First, we validate our quantification method. We used the NREM and REM sleep state-space boundaries that were defined from data collected in period one (i.e., control period ZT3–ZT5) to score NRt bouts in both periods one and two (i.e., the period of muscimol microperfusion in the drug treatment group ZT5.5–ZT7.5). Given that we hypothesized that vlPAG/DpMe inhibition would compromise sleep bistability, resulting in increased NRt time, we tested whether or not our NRt scoring method is predisposed to potential false-positive NRt identification. First, in the group of control rats (*n* = 13), when performing manual 3-stage scoring, Figure 2*A* shows there was no consistent change in the amount of wake, NREM, and REM sleep between period 1 and period 2 expressed as percentages of the total recording time [TRT; paired mean differences (wake = −0.575%, NREM = −0.484%, REM = 1.06%), 95% CIs (wake = −13.2%, 9.68%; NREM = −8.85%, 9.81%; REM = −1.19%, 3.81%)]. Figure 2*B* shows that no spurious changes in sleep-wake state abundance emerged when NRt scoring is performed as there were still no consistent changes in levels of wake, NREM, REM, or NRt between periods 1 and 2 in the control group (paired mean differences (wake = −0.026%, NREM = −0.458%, REM = 0.451%, NRt = 0.320), 95% CIs (wake = −12.3%, 9.97%; NREM = −8.91%, 9.70%; REM = −2.22%, 1.35%; NRt = −0.163%, 0.73%). Figure 2*C* shows that when comparing 3-stage sleep scoring with our 4-stage scoring including NRt in the control group, that NRt epochs were consistently scored as NREM sleep using 3-stage scoring (paired mean difference in NREM for 4-stage scoring minus 3-stage scoring = −3.17% TRT; CI = −4.08%, −2.45%).

### Effects of vlPAG/DpMe inhibition on NREM/REM transitionary sleep

**Figure 3.**
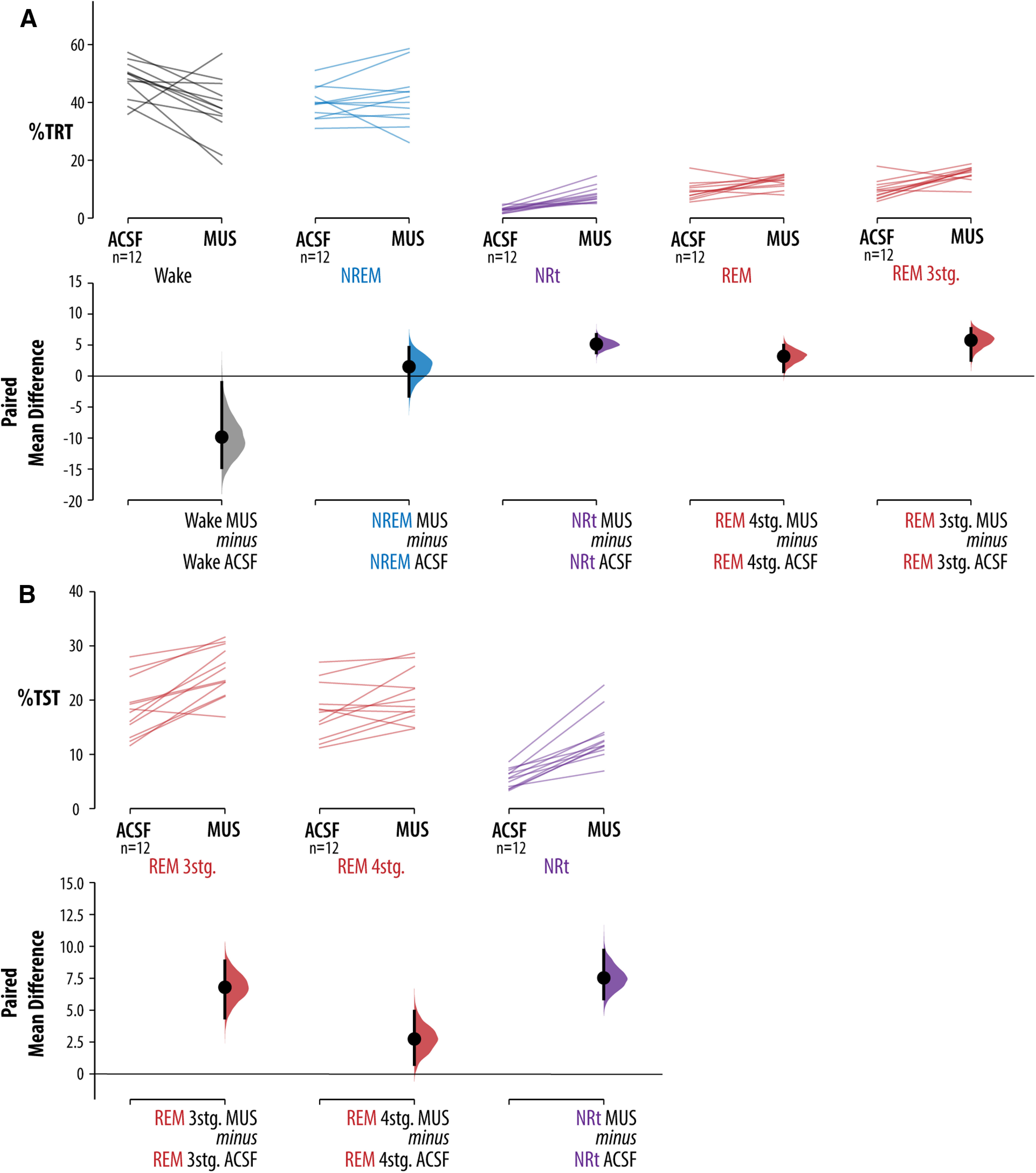
Effects of vlPAG/DpMe inhibition on sleep macroarchitecture and bistability. ***A***, Effects of vlPAG/DpMe inhibition on the levels of sleep-wake states as a proportion of the TRT. Levels of REM sleep are given for both 3-stage scoring and 4-stage NRt scoring. ***B***, Effects of vlPAG/DpMe inhibition on the levels of REM sleep and NRt states as a proportion of the TST. Levels of REM sleep are given for both 3-stage scoring and 4-stage NRt scoring. For each comparison, the paired mean differences are shown in Cumming estimation plots. The raw data are plotted on the upper axes; each paired set of observations is connected by a line. On the lower axes, each paired mean difference is plotted as a bootstrap sampling distribution. Mean differences are depicted as dots; 95% CIs are indicated by the ends of the vertical error bars.

Figure 3*A* shows that muscimol treatment decreased wake, and therefore increased sleep opportunity, by 9.85% of the TRT with a 95% CI of (−14.8%, −1.03%). Note that there is considerable uncertainty about the magnitude of the wake suppression effect, with the CI stretching toward zero difference. Nevertheless, to control for the possibility that inhibition of the vlPAG/DpMe suppresses wake, we chose to quantify changes in sleep and NRt as percentages of the total sleep time (TST). Figure 3*B* shows that, with 3-stage scoring, vlPAG/DpMe inhibition produced a large increase in REM sleep of 6.79% TST with a 95% CI of (4.39%, 8.86%). However, when accounting for changes in sleep bistability using 4-stage NRt scoring, muscimol treatment was associated with a smaller increase in REM sleep of 2.74% TRT [CI (0.748%, 4.92%)], where the CI is compatible with negligible effect sizes. We found that when using 4-stage NRt scoring, much of the observed increase in REM sleep was better classified as bouts of NRt (this despite the evidence detailed in Fig. 2 that our method of 4-stage NRt scoring is not predisposed to reclassification of REM sleep to NRt). Therefore, inhibition of the vlPAG/DpMe compromised normal sleep bistability, as evidenced by a large increase in NRt of 7.53% of the TST with a 95% CI of (5.89%, 9.7%; Fig. 3*B*). The CI here suggests that all compatible effect sizes are large.

### Effects of vlPAG/DpMe inhibition on REM sleep stability

The data presented here after were derived using 4-stage NRt scoring unless otherwise stipulated.

**Figure 4.**
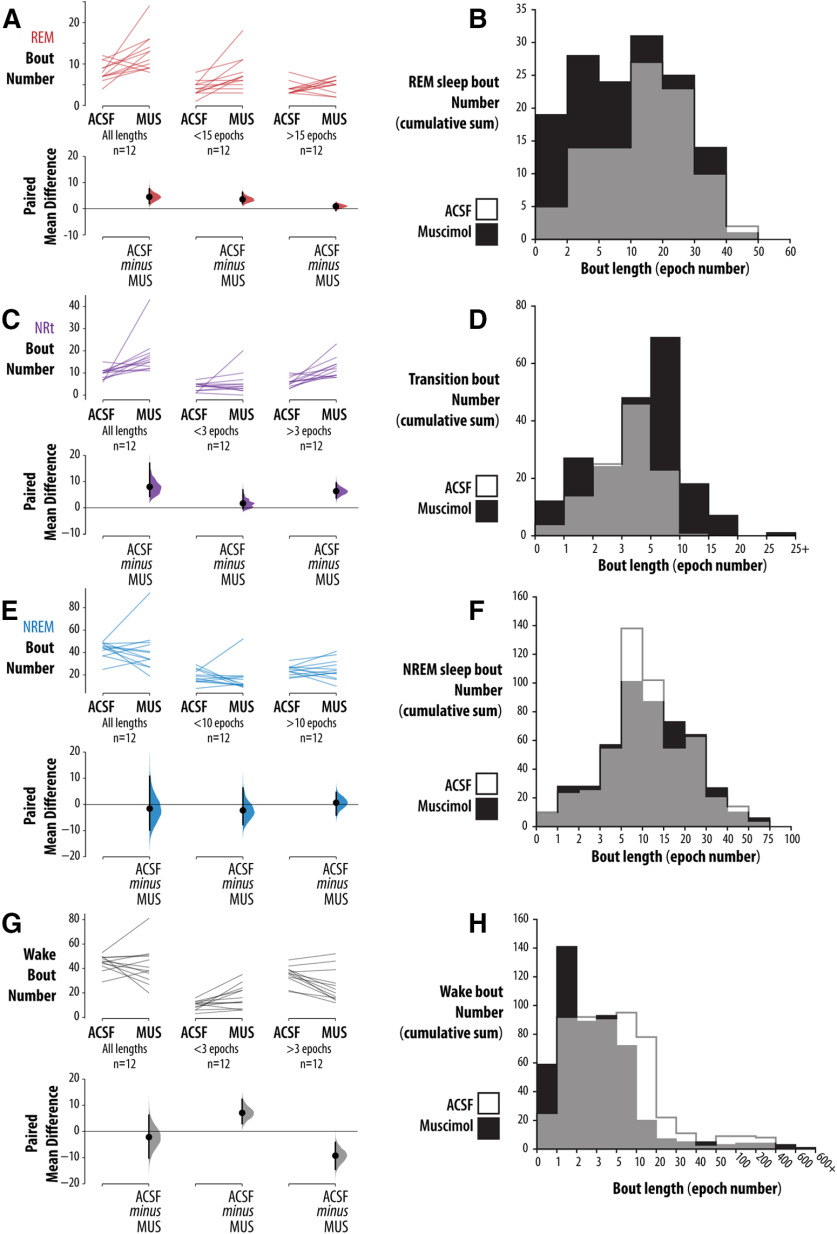
Effects of vlPAG/DpMe inhibition on bout frequency and length. The effects of vlPAG/DpMe inhibition on bout frequency and length for (***A***, ***B***) REM sleep, (***C***, ***D***) NRt, (***E***, ***F***) NREM sleep, and (***G***, ***H***) wake. For each state, we give the total number of bouts, the number of short bouts and the number of long bouts for the ACSF and muscimol conditions (***A***, ***C***, ***E***). For each comparison, the paired mean differences are shown in Cumming estimation plots. The raw data are plotted on the upper axes; each paired set of observations is connected by a line. On the lower axes, each paired mean difference is plotted as a bootstrap sampling distribution. Mean differences are depicted as dots; 95% CIs are indicated by the ends of the vertical error bars. Also, for each state, bout length histograms are provided in ***B***, ***D***, ***F*** showing the cumulative bout numbers across all 12 rats in the muscimol group.

Figure 4*A*,*B* shows that inhibition of the vlPAG/DpMe produced a fragmentation of REM sleep; the average frequency of short (≤15 epochs in duration) REM sleep bouts/2 h increased by 4.5 (an 86% increase relative to baseline) with a 95% CI of (1.92, 6.25). By contrast, the change in longer REM sleep bouts (>15 epochs in duration) was near zero [0.917 bouts/2 h with a CI of (−0.667, 2.0)]. Figure 4*C*,*D* shows that, during muscimol microperfusion animals entered transitional sleep more frequently and remained in NRt longer relative to baseline. The average frequency of long (more than or equal to three epochs in duration) NRt bouts increased by 6.33 bouts/2 h (a 108% increase relative to baseline) with a CI of (3.83, 9.5). Importantly, in contrast to REM sleep, NREM sleep was not consistently fragmented by vlPAG/DpMe inhibition. Figure 4*E*,*F* shows the analysis of NREM sleep bout frequency. For NREM sleep bout number/2 h, the CI of (−9.75, 10.8; mean difference of +1.58 bouts/2 h) is compatible with their being no effect; however, the interval is wide, and so we cannot rule out moderate effect sizes in either direction. The uncertainty largely stems from one animal. Exclusion of this animal yields a CI of (−13.4, 1.0; mean difference of −5.64 bouts/2 h; i.e., a CI that is not compatible with a marked increased NREM sleep bout number). Figure 4 *G*,*H* shows the analysis of waking bout frequencies.

**Figure 5.**
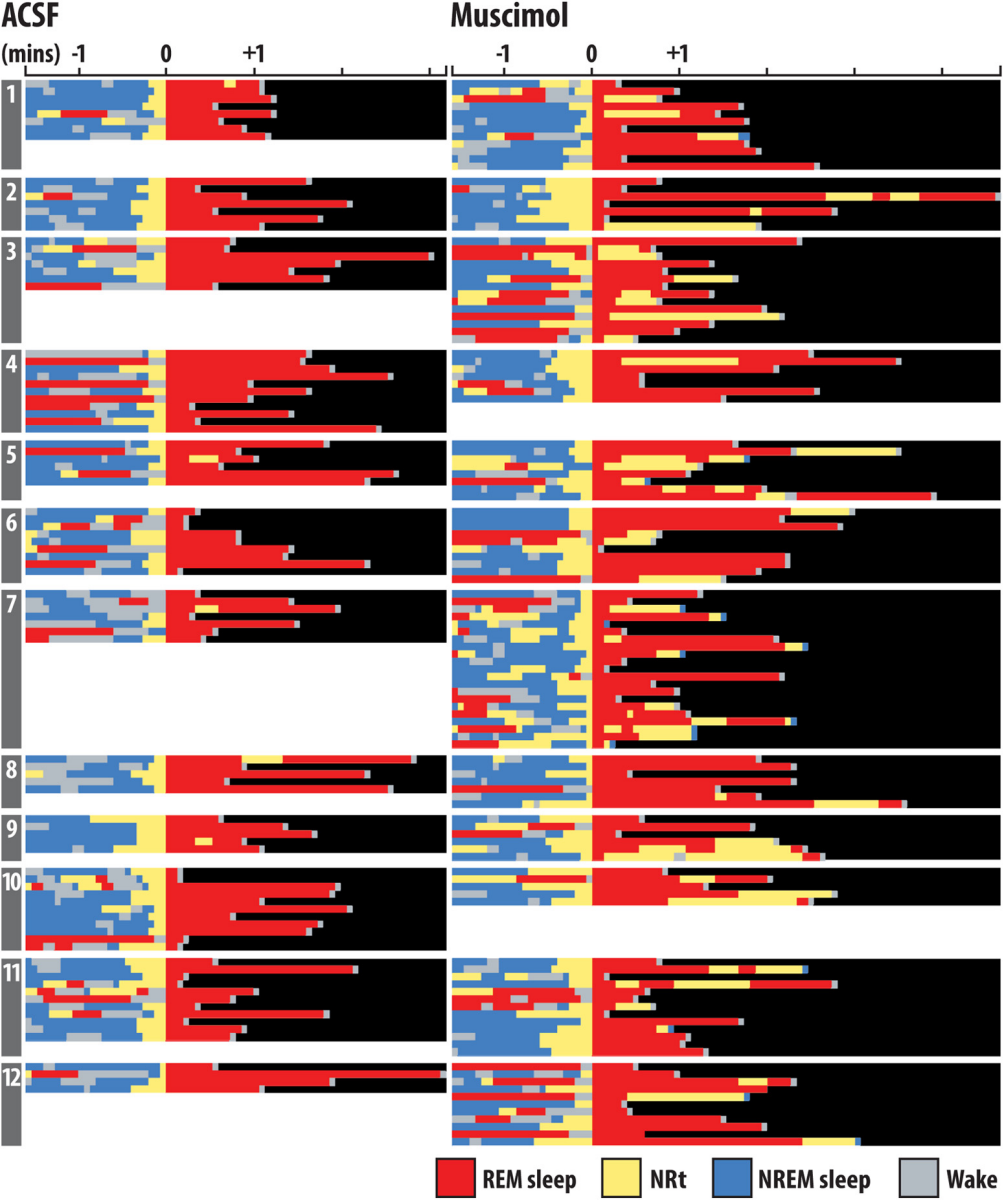
Hypnograms for all transitions into REM sleep. Shown are the hypnograms for all transitions into REM sleep from all rats numbered 1–12, inclusive of REM sleep episodes and the preceding 90 s. All transitions are aligned according to the epoch where the transition into REM sleep takes place (denoted as time 0).

**Figure 6.**
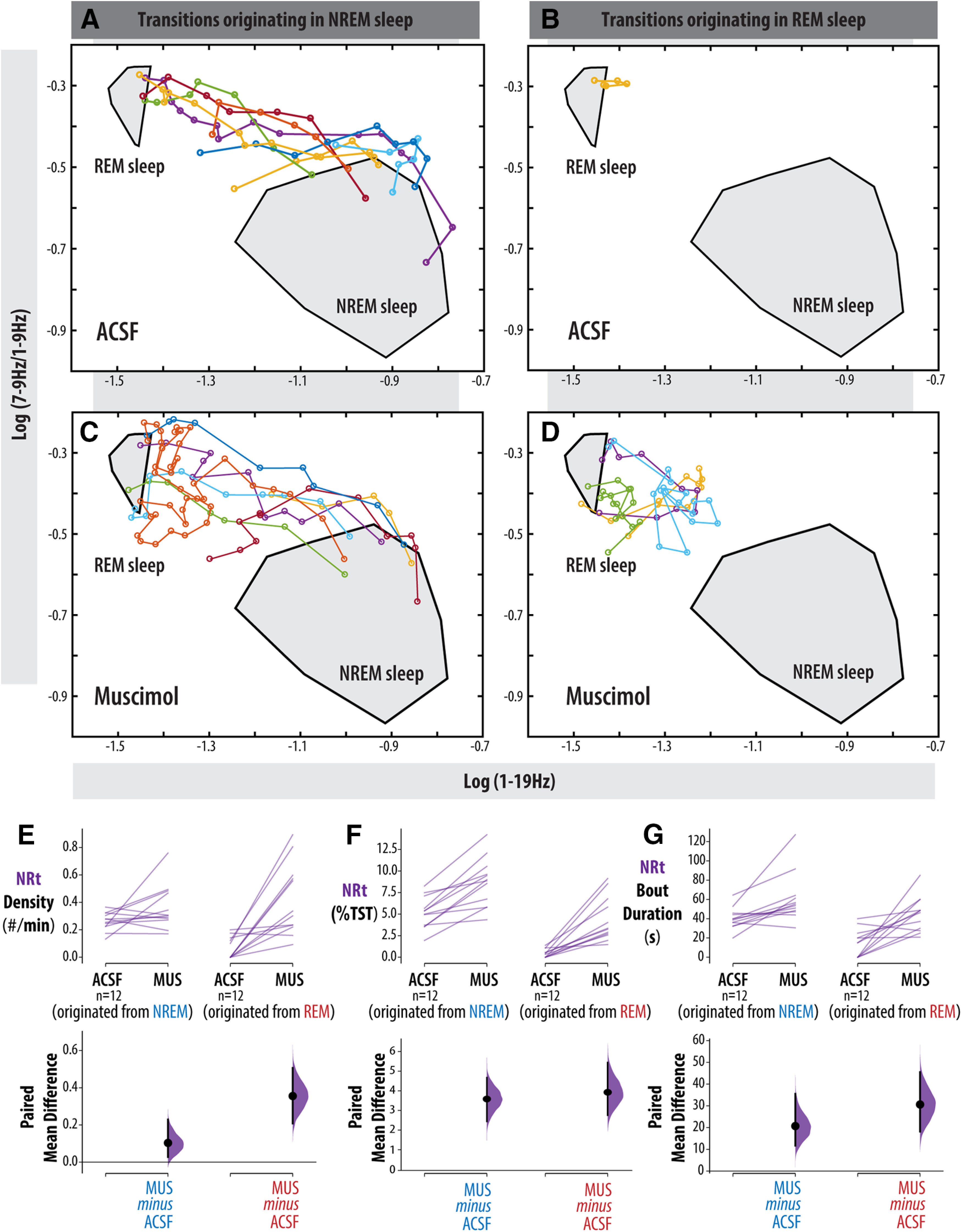
NRt dynamics in NREM versus REM sleep. ***A–D***, Exemplar state-space plot from a single experiment. ***A***, All NRt trajectories originating in NREM sleep space during the baseline period. ***B***, Trajectory of the only excursion into NRT space originating in REM sleep over the same period in the same animal. ***C***, ***D***, Examples of the NRt trajectories originating, from NREM and REM sleep, respectively, during muscimol-mediated inhibition of the vlPAG/DpMe. ***E***, Group data of paired mean difference in NRt density originating in NREM sleep (NRt bouts/minutes of NREM sleep) and REM sleep (NRt bouts/minutes of REM sleep). ***F***, Total NRt amount as a percentage of TST for NRt originating in NREM and REM sleep. ***G***, Average NRt bout duration for NRt originating in NREM and REM sleep. Paired mean differences are shown for all comparisons in a Cumming estimation plot. The raw data are plotted on the upper axes; each paired set of observations is connected by a line. On the lower axes, each paired mean difference is plotted as a bootstrap sampling distribution. Mean differences are depicted as dots; 95% CIs are indicated by the ends of the vertical error bars.

That NREM sleep was not fragmented by muscimol microperfusion suggests that the increased number of NRt bouts induced by vlPAG/DpMe inhibition originated disproportionately from periods of REM sleep. Figure 5 shows the hypnograms for all transitions into REM sleep from all rats in the muscimol group, inclusive of REM sleep episodes and the preceding 90 s. The figure shows that, under normal (ACSF) conditions, entries into NRt from REM sleep were rare, occurring only five times in 24 h of baseline recording in 12 rats. In contrast, during vlPAG/DpMe inhibition there was increased emergence of NRt from REM sleep episodes. Figure 6*A* shows an exemplar state-space plot from a single experiment including all the NRt trajectories originating in NREM sleep space during the baseline period. Figure 6*B* shows the trajectory of the only excursion into NRT space originating in REM sleep over the same period in the same animal. Figure 6*C*,*D* show examples of the NRt trajectories, originating from NREM and REM sleep, respectively, during muscimol-mediated inhibition of the vlPAG/DpMe. Notice in Figure 6*D* the increased propensity to enter NRt from REM sleep during vlPAG/DpMe inhibition. Figure 6*E* gives group data showing that muscimol inhibition increased the density of NRT originating in NREM sleep by 39% (paired mean difference in NRt density (bouts/minute of NREM sleep) = 0.103 [CI (0.027, 0.229)]. However, there is uncertainty about the magnitude of this effect given that the CI is compatible with negligible effect sizes. By contrast, we observed a larger, more robust increase in the density (bouts/minutes of REM sleep) of NRt originating in REM sleep. Muscimol inhibition increased NRt density originating in REM sleep by 576% [paired mean difference in NRt density = 0.355, CI (0.208, 0.506)]. The CI here suggests that all compatible effect sizes are large.

Figure 6*F* shows that while vlPAG/DpMe inhibition may not produce large increases in NREM NRt density, muscimol microperfusion did reliably increase the total amount of NRt originating in NREM sleep. The amount of NRt originating in NREM sleep increased by 3.58% of TST (69.3% increase from baseline) during muscimol microperfusion with a 95% CI of (2.44%, 4.67%). This can be accounted for by an average increase in the duration of NRt bouts originating in NREM sleep of 20.7 s [95% CI (11.7 s, 35.5 s); a 51.5% increase from baseline; Fig. 6*G*]. The amount of NRt originating in REM sleep increased by 3.93% of TST during muscimol microperfusion with a 95% CI of (2.76%, 5.44%), representing an 876% increase relative to baseline (Fig. 6*F*). The duration of NRts originating in REM sleep increased 216% over baseline with a paired mean difference of 30.6 s and a 95% CI of (18.2 s, 45.5 s). Taken together, these data suggest that the loss of sleep bistability resulting from vlPAG/DpMe inhibition was driven by a greater frequency of NRts originating in REM sleep and the lengthening of NRt bouts regardless of the sleep state of origin.

### Effects of vlPAG/DpMe on EEG power

**Figure 7.**
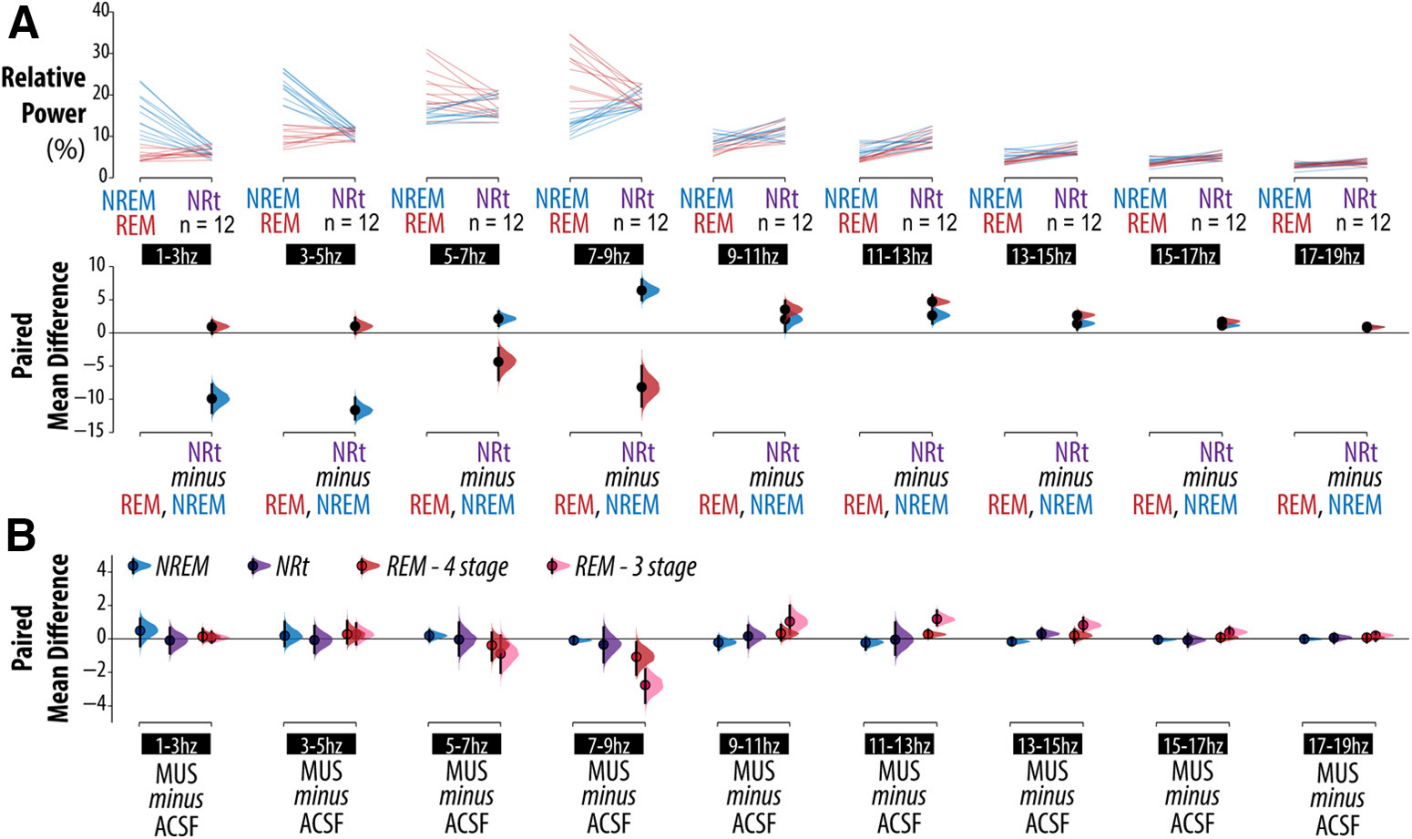
EEG effects of vlPAG/DpMe inhibition. Group data showing the effect of muscimol on relative EEG power (in 2-Hz-wide bins from 1 to 19 Hz). ***A***, Comparison of EEG power between NRt and both NREM and REM sleep in the baseline condition. ***B***, Effect of muscimol inhibition on EEG power for each band and each state (NRt, NREM, and REM sleep). EEG power in REM sleep is given for 3-stage scoring and 4-stage NRt scoring. For each comparison, the paired mean differences are shown in Cumming estimation plots. The raw data are plotted on the upper axes; each paired set of observations is connected by a line. Raw data are omitted in panel ***B***. On the lower axes, each paired mean difference is plotted as a bootstrap sampling distribution. Mean differences are depicted as dots; 95% CIs are indicated by the ends of the vertical error bars. Examples of EEG traces, state-space plots, and NRt trajectories in state space are shown in Extended Data Figure 7-1.

That much of the increase in REM sleep induced by vlPAG/DpMe inhibition is better classified as transitionary sleep, is also evident in the EEG power analysis. Extended Data Figure 7–1*A–D* shows an example of spectral changes in the EEG across a normal NREM-to-REM sleep transition (Extended Data Fig. 7–1*A*,*B*) compared with the spectral changes across a period of sleep instability during vlPAG/DpMe inhibition (Extended Data Fig. 7–1*C*,*D*) with corresponding state-space plots showing the state trajectories in NRt space. Figure 7*A* shows baseline group data comparing the difference in relative EEG power, across multiple frequency bands, between: (1) NREM sleep and NRt (blue lines), and (2) REM sleep and NRt (red lines). Figure 7*A*, bottom panel of the Cumming estimation plot, shows the frequency ranges where NRt, NREM, and REM sleep states reliably differ from one another. At baseline, relative to NREM sleep, NRt periods exhibited reduced δ power [1–3 Hz; CI (−12.0, −7.77)], elevated θ power [7–9 Hz; CI (4.94, 8.05)], and elevated σ power [11–13 Hz; CI (1.41, 3.86)]. Relative to REM sleep, NRt periods exhibited reduced θ power [7–9 Hz; CI (−11.1, −4.97)] and elevated σ power [11–13 Hz; CI (4.04, 5.69)]. Figure 7*B* shows the effect of vlPAG/DpMe inhibition on relative EEG power. For each power band, we show the bootstrap sampling distributions of the paired mean differences for each state (data points from individual animals are omitted for clarity). We found that the largest and most robust effects on EEG power occurred in REM sleep, specifically in those frequency bands where REM sleep differed most from NRt, i.e., 7–9 and 11–13 Hz. The magnitude of power changes in these bands was greatest when using 3-stage scoring. The CIs suggest that all compatible effect sizes are large. When changes in sleep bistabilty were accounted for with 4-stage NRt scoring, the effect sizes of muscimol inhibition on EEG power changes in REM sleep were diminished and the CIs are, instead, compatible with smaller or negligible effect sizes. The attenuation of EEG power effects in REM sleep, when accounting for the incidence of NRt bouts, indicates that these power differences stemmed from these discrete NRt bouts rather than a more general modulation of REM sleep EEG features.

10.1523/ENEURO.0451-19.2020.f7-1 Extended Data Figure 7-1 Examples of normal and unstable EEG dynamics across transitions between NREM and REM sleep. ***A***, Example spectrogram and corresponding state-space plot depicting normal spectral changes in the EEG across a NREM-to-REM sleep transition during ACSF microperfusion of the vlPAG/DpMe. ***B***, The corresponding state-space plot for the EEG and EMG traces shown in ***A***. The state-space trajectory moves: (i) from NREM sleep (one blue dot per epoch within NREM sleep boundary), (ii) into transitionary space (one black arrow per epoch; arrow direction indicating the direction of the state-space trajectory), (iii) and finishes within the REM sleep boundary. ***C***, Example spectrogram and corresponding state-space plot depicting a period of unstable sleep during muscimol-mediated inhibition of the vlPAG/DpMe. ***D***, The corresponding state-space plot for the EEG and EMG traces shown in ***C***. The colors for the state trajectory segments correspond to the hypnogram colors in ***C***. The trajectory begins in NREM sleep at point 1, moves into transitionary space during epoch 8, moves into REM sleep space at epoch 13, completes a transition from REM to NREM sleep space at epoch 15, moves back into transitionary space during epoch 21, re-enters NREM sleep space at epoch 30 having failed to enter REM sleep space, remains in NREM until epoch 31 before re-entering transition space and completing a transition into REM sleep space at epoch 36. Download Figure 7-1, TIF file.

### Computer simulations of flip-flop circuits: optimizing baseline flip-flop behavior

**Figure 8.**
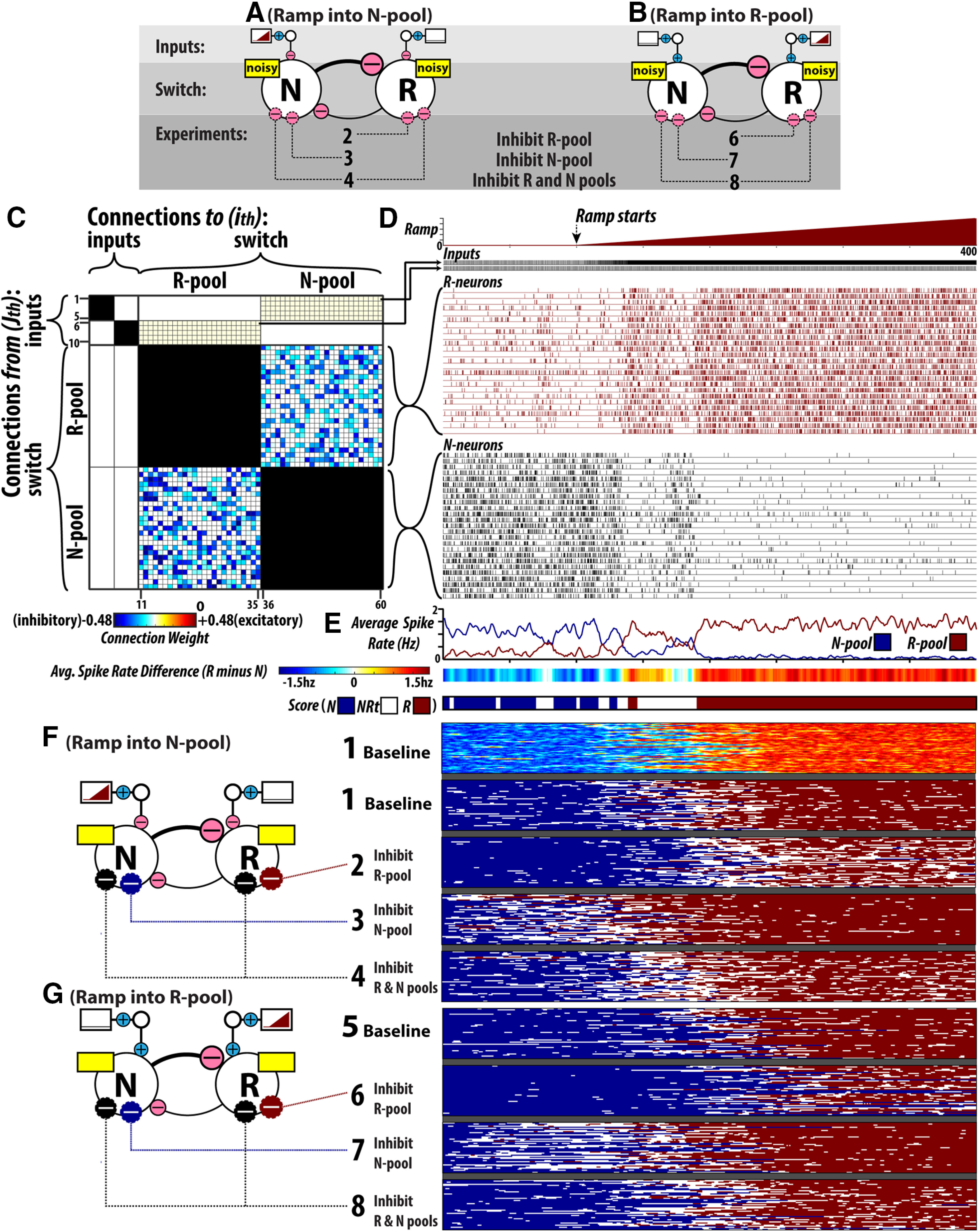
Computer Simulations of flip-flop circuits. ***A***, ***B***, Configurations of the simulated flip-flop circuits. Two input scenarios are used: (***A***) ramping R-state drive is delivered to the flip-flop switch through the N-pool and (***B***) ramping R-state drive is delivered to the flip-flop switch through the R-pool. ***C***, Connectivity matrix for one example simulation circuit. Each cell in the grid corresponds to the weight of a synaptic connection. Each row corresponds to an individual neuron as a source of connections, while every column corresponds to an individual neuron as a recipient of input. Neurons are grouped according to the pools to which they belong: input pool (*n* = 10), R-pool (*n* = 25), and N-pool (*n* = 25). The regions of the matrix filled with black denote the fact that there is no intrapool connectivity. Otherwise, the fill color of the cells denotes the synaptic weight between cells (white denotes a weight of zero or no connection). N→R and N→R inhibitory connections depicted are only representative examples (47 circuits were used across all simulations, where the N→R and N→R inhibitory connections were randomized). ***D***, An example simulation run. Spike raster plots are shown for representative R-pool and N-pool input neurons (every fifth input neuron spike is shown to make visualizing the spikes easier). Spike raster plots are also shown for all N-pool and R-pool neurons. ***E***, N-pool and R-pool raster plots as average population levels over the time course of the simulation, average spike rate difference between the pools (R-pool minus N-pool spiking), and a corresponding scoring bar, where spike rate differences are converted to one of three states: N, NRt, and R. ***F***, ***G***, Shows the results of every simulation across all experimental conditions. Simulation results are presented in the state-scoring format depicted in ***E***. The procedure for setting flip-flop synaptic weights and excitatory bias currents is illustrated in Extended Data Figure 8-2. A listing of the parameter values/settings used for computer simulations is given in Extended Data Table 8-1.

We used neural circuit simulations to evaluate whether the effects, on NRt dynamics in particular, of vlPAG/DpMe inhibition *in vivo* are consistent with the predicted changes in NRt dynamics resulting from modulation of an underlying flip-flop circuit. We simulated a ramping input signal into a flip-flop switch composed of two pools of noisy, tonically active LIF-type neurons that are mutually inhibitory, randomly connected, and asymmetrically weighted (for more detail, see Materials and Methods). Figure 8*A*,*B* shows the configurations of the simulated flip-flop circuits. It is important to keep in mind that we consider two input scenarios: one where the ramping R-state drive input is delivered to the flip-flop switch through the stronger N-pool (Fig. 8*A*) and one where it is delivered through the weaker R-pool (Fig. 8*B*). Figure 8*C* gives the connectivity matrix for one example simulation circuit. Each cell in the grid corresponds to the weight of a synaptic connection. Each row corresponds to an individual neuron as a source of connections, while every column corresponds to an individual neuron as a recipient of input. Neurons are grouped according to the pools to which they belong: input pool (*n* = 10), R-pool (*n* = 25), and N-pool (*n* = 25). The regions of the matrix filled with black denote the fact that there is no intrapool connectivity. Otherwise, the fill color of the cells denotes the synaptic weight between cells (white denotes a weight of zero or no connection). It is important to keep in mind that the N-pool→R-pool and R-pool →N-pool inhibitory connections depicted in Figure 8*C* are only examples; we constructed 47 circuits where the N-pool→R-pool and R-pool→N-pool inhibitory connections were randomized. An example simulation run is shown in Figure 8*D*. In the case of this example, the ramping input is delivered through the N-pool. Spike raster plots are shown for representative R-pool and N-pool input neurons. Spike raster plots are also shown for all N-pool and R-pool neurons. Figure 8*E* gives the average population levels of firing for the N-pool and R-pool over the time course of the simulation. From the population activities, we calculated the average spike rate difference between the pools (R-pool minus N-pool spiking). Average spike rate difference is represented as a color-coded bar in the middle segment of Figure 8*E* and a matching “scoring” bar, where spike rate differences are converted to one of three states: N, NRt, and R (according to the scoring rules stipulated in Materials and Methods, Technical details of flip-flop circuit computer simulations).

10.1523/ENEURO.0451-19.2020.t8-1 Extended Data Table 8-1 Parameters used for computer simulations of flip-flop circuits. A listing of all the parameters used to produce the flip-flop circuit simulations. Download Table 8-1, DOCX file.

The baseline behavior of the flip-flop circuits used in these simulations is very sensitive to the balance of the synaptic weighting between N-pool and R-pool and the level of excitatory bias current applied to the neurons. We examined flip-flop switch behavior across a range of N-pool and R-pool weighting combinations and excitatory bias currents in the absence of any ramping R-state drive. The results of those simulations are shown in Extended Data Figure 8-2. As outlined in Materials and Methods (Setting flip-flop synaptic weights and excitatory bias currents), for each weighting combination, we performed 25 simulations, calculated the average spike rate difference between the N-pool and R-pool and created a histogram showing the frequencies of binned R-pool minus N-pool firing rate differences. Examples of such histograms are shown in Extended Data Figure 8-2*B–D*. In these histograms, the magnitude of the left and right peaks indicates the prevalence of the N-state and R-state, respectively. The height of the intervening trough indicates the prevalence of N/R intermediate states. In Extended Data Figure 8–2*E*,*F*, individual cells in the grid correspond to a given parameter combination and the color-coding is a representation of the associated firing rate histogram: the height of the N-peak, N/R trough, and the R-peak are indicated by the color of the left, middle and right bars, respectively. The parameter combinations selected for the experimental simulations are outlined in yellow. We chose a combination of R→N and N→R connection weights where N→R weights are slightly stronger. This results in dominance of the N-state while maintaining the tendency of the circuit to periodically transition into an R-state in the absence of any ramping R-state drive input. Without a modest tendency to switch into R-states despite the lack of switching input (a result of including noise in the bias currents), we do not observe “failed” transition attempts (i.e., N→NRt→N or R→NRt→R events) as occur *in vivo*.

10.1523/ENEURO.0451-19.2020.f8-2 Extended Data Figure 8-2 Computer Simulations of flip-flop circuits: setting flip-flop synaptic weights and excitatory bias currents. ***A***, Connection weighting was tuned prior to setting the level of excitatory bias current. Initial connection weights were divided by a factor, *d*, ranging from 1.8 to 2.8 in 0.1-unit increments. N_j_→R_i_ weights were changed independent of R_j_→N_i_ weights. A total of 121 combinations of N_j_→R_i_ of and R_j_→N_i_ weighting were used (Extended Data Fig. 9-1*A*). ***B–D***, Examples (3/121) of the simulated combinations of flip-flop weighting. For each weighting combination, we calculated the difference in population firing rate over time and converted these data to a histogram showing the frequencies of binned R-N firing rate differences. Bimodal histograms indicate flip-flops that spontaneously switch between N-state and R-state. The height of the left and right peaks of the histograms indicate the prevalence of the N-state and R-state, respectively. The height of the intervening trough indicates the prevalence of N/R intermediate states. ***E***, ***F***, 11 × 11 simulation spaces where individual cells in the grid correspond to a given parameter combination and the color coding is a representation of the firing rate histogram generated from that particular set of simulations: the height of the N-peak, N/R trough, and the R-peak are indicated by the color of the left, middle, and right bars, respectively. The parameter combinations selected for experimental simulations are outlined in yellow. ***E***, The stimulation space for synaptic weight (columns correspond to R→N weighting; rows correspond to N→R weighting). ***F***, The simulation space for excitatory bias current (columns correspond to R neuron current; columns correspond to N neuron current). Download Figure 8-2, TIF file.

The effect of any inactivation or activation of R or N neurons is equivalent to movement between cells of the grid in Extended Data Figure 8-2*F*. For instance, when reducing the excitability of N-pool and R-pool simultaneously, equivalent to moving toward the top left corner of the grid, flip-flop circuits lose bistability, as evidenced by the increased time spent in NRt. It is important to note that given the nonlinear changes in flip-flop switch behavior across bias current space, that the qualitative nature of the effect of any change in N-pool and/or R-pool activation level will be sensitive to the initial configuration of the switch.

### Computer simulations of flip-flop circuits: the effects of neuronal inhibition on switching behavior and bistability

**Figure 9.**
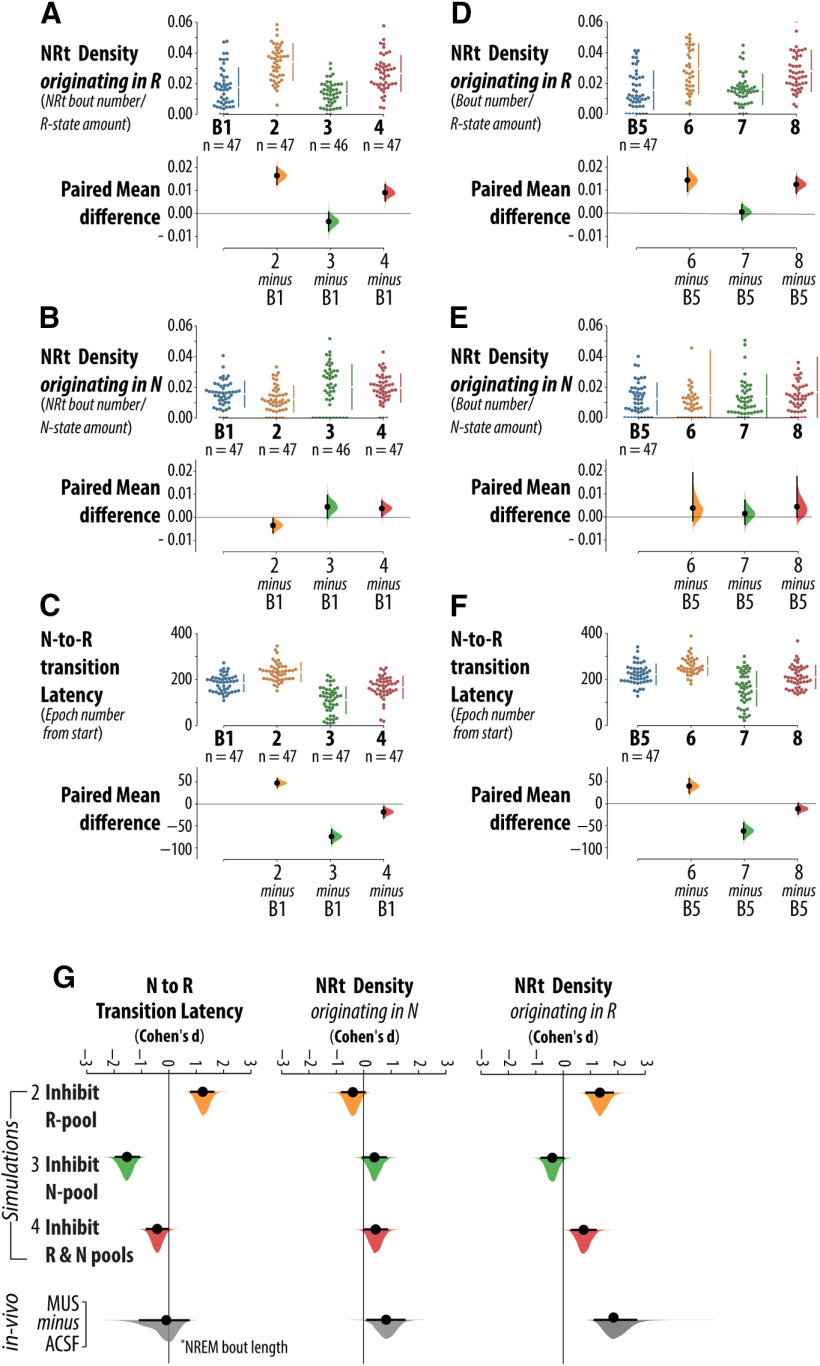
Computer simulations of flip-flop circuits: analysis of bistability and switching behavior. ***A***, Group data for NRt density originating in the R-state, calculated ratio of the number of NRt bouts over the amount of R-state, across four sets of simulations: B1 (baseline), 2 (inhibition of the R-pool), 3 (inhibition of the N-pool), and 4 (inhibition of the N-pool and R-pool). ***B***, NRt density originating in the N-state, calculated ratio of the number of NRt bouts over the amount of N-state, for simulation sets (B1, 2, 3, and 4). ***C***, Latency to the R-state for simulation sets (B1, 2, 3, and 4). For each comparison, the paired mean differences are shown in Cumming estimation plots. The raw data are plotted on the upper axes. On the lower axes, each paired mean difference is plotted as a bootstrap sampling distribution. Mean differences are depicted as dots; 95% CIs are indicated by the ends of the vertical error bars. ***D–F***, The results of simulations using circuits where the ramping input was delivered through the R-pool (B5, 6, 7, and 8). ***G***, Compares the effects from our *in vivo* dataset with that from simulated experiments. Effect sizes are standardized (Cohen’s *d*). Simulation sets (B1, 2, 3, and 4) use the circuit configuration where the ramping input is delivered to the flip-flop through the N-pool.

Figure 8*F*,*G* show the results of every simulation across all experimental conditions. Simulation results are presented in the “state-scoring format” depicted in Figure 8*E*. Note that Figure 8*F* also shows the average spike rate difference between N-pool and R-pool, in the colored bar format, for all B1, baseline simulations. By selectively reducing the applied currents to neurons in the N-pool and R-pool, we performed three experiments: inhibition of the R-pool, inhibition of the N-pool and combined inhibition of the N-pool and R-pool. This is in keeping with the possible effects of vlPAG/DpMe inhibition *in vivo*, as this region is enriched in both REM sleep-inactive and REM sleep-active neuron types. We used several measures to quantify flip-flop switch behavior that are based on our *in vivo* analyses: the density of NRt bouts originating in the R-state, the density of NRt bouts originating in the N-state, and the latency of transition to the R-state (simulations are initialized in the N-state). Quantification of the simulation results according to these metrics is presented in Figure 9.

Figure 9*A* shows the results of experimental simulations on the density of NRt bouts originating in the R-state (i.e., the frequency of NRt bouts relative to the total number of R-state epochs). Relative to the baseline configuration, inhibition of the R-pool (simulation set 2) robustly increased the frequency of NRt originating in the R-state by 93% with a 95% CI of (74%, 116%). By contrast, inhibition of the N-pool (simulation set 3) did not increase the frequency of NRt originating in the R-state (paired mean difference −24% with a 95% CI of (−43%, 2%). In simulation set 4, where both N-pool and R-pool were inactivated, the frequency of NRt originating in the R-state increased 51% with a 95% confidence in interval of (33%, 73%). Therefore, the CIs indicate that, a marked increase in R-state NRt density is only compatible with R-pool and combined R-pool/N-pool inhibition.

Figure 9*B* shows the results of experimental simulations on the density of NRt bouts originating in the N-state (i.e., the frequency of NRt bouts relative to the total number of R-state epochs). Relative to the effect sizes in Figure 9*A*, showing the propensity of the R-state to be destabilized and fragmented by NRt, the CIs shown in Figure 9*B* indicate that the N-state was associated with much smaller, possibly negligible, changes in average NRt density. Relative to the baseline configuration, inhibition of the R-pool (simulation set 2) decreased the frequency of NRt originating in the N-state by 21% with a 95% CI of (−43%, −3%). This is in contrast to the robust increase in NRt density in the R-state occurring in the same simulations. Inhibition of the N-pool (simulation set 3) yielded a CI of N-state NRt effect size that is compatible with no effect (−2%, 61%; paired mean difference = +30%). However, the interval is wide and therefore admits of moderate increases in the frequency of NRt originating in the N-state with N-pool inhibition. A similar result was obtained in experiment 4, where both N-pool and R-pool were inactivated. Here, the frequency of NRt originating in the N-state increased 24% with a 95% confidence in interval of (4%, 46%).

Figure 9*C* shows the results of experimental simulations on the latency to initiation of the R-state (i.e., the drive threshold for N-to-R-state transitioning). Relative to the baseline configuration, inhibition of the R-pool (experiment set 2) robustly increased R-state latency by 26% with a 95% CI of (20%, 31%). In contrast, inhibition of the N-pool (experiment 3) robustly decreased R-state latency by 40% with a 95% CI of (−49%, −32%). In experiment 4, inactivation of both N-pool and R-pool produced a markedly smaller 10% decrease in R-state latency with a 95% confidence in interval of (−17%, −4%).

The preceding results are from stimulations of circuit configurations where the ramping input was delivered through the N-pool (i.e., simulations 1–4). The results of experiments 5–8, where the ramping input was delivered through the weaker R-pool, were not markedly different. Group data for simulations 5–8 are included in Figure 9*D-F*.

Figure 9*G* summarizes the results of vlPAG/DpMe inhibition *in vivo* and that of simulated flip-flop switch inactivations. Standardized effect sizes (Cohen’s *d*) are given for: (1) NRt density originating in R/REM, (2) NRt density originating in N/NREM, and (3) the latency to R/REM, which is represented by NREM sleep bout length in the case of *in vivo* data. The major effects of simulated inhibition of the R-pool were an increase in NRt density originating in the R-state and an increase in the latency to initiation of the R-state. While the fragmentation of the R-state by NRt is consistent with the fragmentation of REM sleep by NRt *in vivo*, the increase in R-state latency is not consistent with our *in vivo* findings. The major effects of simulated inhibition of the N-pool were mostly inconsistent with our *in vivo* findings, which is notable considering that the prevailing hypothesis of vlPAG/DpMe involvement in REM sleep control focuses on the role of REM sleep-inactive neurons in gating NREM-to-REM sleep transitioning. The major effect of simulated N-pool inactivation was reduced R-state transition latency. To be consistent with this scenario, we would have expected shortening of NREM sleep bout lengths *in vivo*, indicating a lowered switching threshold for NREM-to-REM sleep transitioning. Most importantly, simulated N-pool inactivation failed to increase NRt density originating in the R-state. This is not compatible with the largest effect of vlPAG/DpMe inhibition *in vivo*. The *in vivo* effects of vlPAG/DpMe inhibition are most compatible with the scenario where both vlPAG/DpMe REM sleep active and inactive populations contribute to REM sleep regulation through participation in a flip-flop switch. Inhibition of both N-pool and R-pool in flip-flop simulations was necessary to produce increased NRt density originating in the R-state alongside smaller or negligible effects on N-state NRt density and the threshold for N-to-R-state transitioning.

## Discussion

The results of our GABAergic inactivation of the vlPAG/DpMe are consistent with the REM sleep enhancement reported by previous inactivation studies (Petitjean et al., 1975; Sastre et al., 1996; Crochet et al., 2006; Lu et al., 2006a; Vanini, et al., 2007; Sapin et al., 2009; Weber et al., 2015, 2018; Hayashi, 2015). Based on these reported increases in REM sleep following vlPAG/DpMe inactivation, it is reasonable that current hypotheses of vlPAG/DpMe involvement in REM sleep control are focused on the role of REM sleep-inactive neurons in gating NREM-to-REM sleep transitioning (Lu et al., 2006a; Sapin et al., 2009; Saper et al., 2010). However, after accounting for changes in sleep bistability using an analysis of NRt dynamics (both *in vivo* and in the context of flop-flop circuit simulations), we find that current thinking is too narrowly focused on the role of the REM sleep-inactive population of the vlPAG/DpMe in the control of REM sleep.

We argue that the narrow focus on REM sleep-inactive vlPAG/DpMe neurons may be the result of a narrow focus on REM sleep bout statistics as an output measure. Our results show that a portion of the reported increase in REM sleep following vlPAG/DpMe inhibition may be more appropriately classified as a NREM/REM intermediate state (NRt), indicating that some vlPAG/DpMe neurons participate in a sleep bistability mechanism. Small amounts of NRt are a feature of normal sleep cycling (Benington et al., 1994). Episodes of REM sleep are commonly preceded by transitionary periods where the EEG signatures of NREM sleep evolve into that of REM sleep while the discharge rates of REM sleep-active brainstem neurons gradually increase from low rates in NREM sleep to maximal firing rates in REM sleep (Steriade and McCarley, 2005; Boucetta et al., 2014; Weber et al., 2018). The small proportion of time spent in NRt, <5% of the total sleep-wake record, is evidence of its instability relative to NREM sleep and REM sleep states. On this basis, it is reasonable that many researchers do not give NRt major consideration when describing experimental manipulations of sleep-state dynamics.

However, in the case of vlPAG/DpMe neuronal activation, we found an increase in NRt to be the most robust effect. Importantly, NRt periods predominately originated in REM sleep. Under normal conditions, entries into NRt from REM sleep were rare. This fragmentation of REM sleep episodes by NRt bouts resulted in an increase in the number of short REM sleep bouts. With conventional sleep staging, an episode of REM sleep fragmented by NRt can be scored either as a continuous bout or as multiple transitions between REM sleep and NREM sleep. In the former case, frequent transitioning between REM sleep and NRt will present as a shift in the distribution of REM sleep bout lengths toward longer episodes, rather than a fragmentation of the REM sleep state. This is important because previous vlPAG/DpMe inactivation studies, which did not quantify NRt periods, have reported lengthening of REM sleep bouts (Sastre et al., 1996; Crochet et al., 2006; Lu et al., 2006a; Weber et al., 2015). In the latter case, frequent transitioning between REM sleep and NRt will present as a fragmentation of both REM sleep and NREM sleep bout lengths. These scoring variations can produce major differences in functional interpretation from the same data. Therefore, in cases where experimental manipulations may compromise sleep bistability, we suggest that transitionary states be quantified.

In the scenario that vlPAG/DpMe REM sleep-inactive neurons contribute to negatively regulating the initiation of REM sleep, while neighboring REM sleep-active neurons are not significant contributors to REM sleep generation, we would expect specific changes to result from vlPAG/DpMe inhibition. Suppression of the vlPAG/DpMe would be expected to increase the probability of transitioning from NREM-to-REM sleep as evidenced by a shortening of NREM sleep bout lengths and an increase in the number of transitions from NREM-to-REM sleep and/or NRt. However, we did not observe a shortening of NREM sleep bout lengths. Moreover, simulated inactivation of the flip-flop N-pool failed to destabilize and fragment the R-state with bouts of NRt. In fact, N-pool inactivation was compatible with a moderate stabilization of the R-state. This prediction contradicts our *in vivo* finding that REM sleep was fragmented by NRt during vlPAG/DpMe inhibition. In contrast, simulated inhibition of the REM sleep-active component of a flip-flop switch produced a strong and disproportionate fragmentation of REM sleep by NRt. However, the scenario where vlPAG/DpMe REM sleep-active neurons exert major control over the stability of REM sleep while REM sleep-inactive neurons contribute negligible functional output is also not in agreement with our *in vivo* experimental data. Simulated inhibition of the REM sleep-active component of a flip-flop switch delayed transitioning in to REM sleep, but we did not find a decrease in REM sleep propensity *in vivo*.

Our *in vivo* findings are most consistent with the results of simulated suppression of both NREM and REM sleep-active flip-flop switch components. While inhibition of the individual N-pool and R-pool led to decreased and increased N-state propensity, respectively, the combined suppression of both pools resulted in a preservation of transition latency/threshold. This result is important because it is consistent with our *in vivo* finding where, despite a loss of sleep bistability, the distribution of NREM sleep bout lengths was largely unchanged by vlPAG/DpMe inhibition (a shift in NREM sleep bout lengths toward shorter or longer durations would be an indicator of an increase or decrease, respectively, in NREM-to-REM sleep transition propensity). In the context of a flip-flop mechanism of bistability, such changes in propensity reflect changes in the threshold sensitivity of the switch to the level of external drive. Our simulations indicate that this threshold is controlled by both the REM sleep active and inactive switch components, as both pools contribute to the overall strength of positive feedback in the switch. Therefore, while our *in vivo* data seems to suggest that vlPAG/DpMe inhibition did not produce a net change in the threshold sensitivity of the NREM/REM switching circuitry, this is wholly consistent with combined inhibition of mutually inhibitory NREM and REM sleep active switch components.

In contrast to the dependence of switching threshold on both NREM and REM-sleep active switch components, our simulations suggest that the robustness of the flip-flop switch to noise/variability is primarily determined by the weaker REM sleep-active pool. Without inclusion of noise sources, switching between states in our flip-flop simulations would be unidirectional, irreversible and solely dependent on input from the external drive. Previous modeling studies show that flip-flop circuits, with appropriately tuned parameters are capable of reproducing the temporal organization of rodent and human sleep only where source(s) of variability and noise are incorporated (Rempe et al., 2010; Kumar et al., 2012; Dunmyre et al., 2014). On a short time scale (epoch-by-epoch), sleep-wake state dynamics can be treated as a Markov process where the probability of state transitioning is only dependent on the current state of the system and therefore independent of past states (Zung et al., 1965; Yang and Hursch, 1973; Kemp and Kamphuisen, 1986; Gregory and Cabeza, 2002; Diniz Behn et al., 2007; Bianchi et al., 2012; Stephenson et al., 2013, 2016). Over comparatively longer time scales, this property breaks down and higher order dependencies emerge in state dynamics. While it is unlikely that short-term sleep state dynamics strictly conform to the properties of a first-order Markov process (i.e., complete “memorylessness”), it is nevertheless useful to explain sleep patterning in terms of interactions between short-term stochastic processes and long-term processes, including sleep propensity drives. Therefore, it is important that models of sleep state switching, such as our own, incorporate both deterministic and stochastic sources of switching. The aim of previous modeling studies has been to show proof of concept that individual or coupled flip-flop switches are plausible circuit arrangements underling sleep state transitioning (Rempe et al., 2010; Kumar et al., 2012; Dunmyre et al., 2014). However, past studies have not explicitly modelled NREM/REM intermediary states. Here, our primary aim was to simulate changes in NRt dynamics in response to different flip-flop switch configurations and simulated inhibition of switch components. Previous modeling has shown that weakening input to a flip-flop switch will have the effect of increasing the frequency of transitioning between steady states (Rempe et al., 2010). In our simulations, when the parameters are appropriately tuned, flip-flop switches are guaranteed to transition between N-state and R-state at least once under the influence of the external R-state promoting input. Additional transitioning (i.e., N→NRt→N and R→NRt→R transitions) is strictly driven by sources of noise and therefore this type of transitioning reflects robustness of the switching circuit to sources of noise and variability. We found that when a flip-flop switch is asymmetrically weighted (i.e., N-pool→R-pool inhibition dominates), the weaker pool dictates switch robustness to noise and the switch’s sensitivity to noise is strongest when activity in the weaker pool is highest. Therefore, based on our simulations, the disproportionate fragmentation of REM sleep by NRt, that we observed *in vivo* following vlPAG/DpMe inhibition, is consistent with an inhibition of the REM sleep-active subpopulation of the vlPAG/DpMe.

In our simulations, we did not include negative feedback between the switch output and the REM promoting drive (i.e., a homeostatic control circuit). Homeostasis and bistability, while related, are nevertheless separable features of the REM sleep control circuitry. Our study focuses on sleep bistability per se, and on what NRt dynamics reveal about the underlying mechanism of bistability. Future modeling studies are needed that incorporate predictions of NRt bout statistics in the context of interacting NREM and REM sleep homeostats. For such models, it will be important to consider the influence of NRt on the dissipation of REM sleep and NREM sleep drives. Past REM sleep deprivation studies have shown that NRt propensity seems to increase over the course of selective REM sleep deprivation, but that NRt per se does not exhibit rebound following deprivation (Maloney et al., 1999, 2000). Moreover, the occurrence of REM sleep rebound following deprivation suggests that the increased prevalence of NRt during deprivation is insufficient for the dissipation of REM sleep drive.

It is important to note that because we do not model REM sleep homeostasis that the ramping R-state drive continues to increase in the R-state in our simulations. In vivo, we would hypothesize that REM sleep drive would dissipate in association with REM sleep bouts. However, we currently do not know what the dynamics of this drive are on the order of individual epochs and bouts of REM sleep. Nevertheless, it is important to address what influence the drive signal profile during the R-state has on flip-flop switch behavior. In the simulations, NRt bouts are, by definition, periods where N-pool and R-pool exhibit intermediate firing rates. Once the R-state is reached, further increases in the ramping drive, that promote R-pool firing and inhibit N-pool firing, tend to stabilize the R-state, i.e., reduces the likelihood of intermediate firing rates in N-pool and R-pool. Therefore, the choice to use a monotonically increasing linear ramp can be considered conservative, as it biases flip-flip switch behavior away from exhibiting NRt in the R-state.

We considered two different flip-flop circuit configurations: one where the R-state promoting drive inhibited the stronger N-pool and one where that drive activated the weaker R-pool. In our simulations, we did not detect major differences in flip-flop switch behavior that were sensitive to drive input pathway (i.e., the integration site for REM sleep drive). Nevertheless, more studies are needed to identify the populations that integrate REM sleep drive signals and to characterize the nature of the drive signals themselves. Weber et al. (2018), reported that firing rate increases exhibited by REM sleep-active vlPAG neurons before transitions into REM sleep are not significantly different from those increases that precede transitions into wake. By contrast, decreases in NREM sleep-active (i.e., neurons exhibiting greater firing rates in NREM sleep than REM sleep, but not necessarily maximal firing rates in NREM sleep) vlPAG neuron firing in advance of REM sleep episodes are greater in magnitude compared with those that precede transitions into wake. This suggests that the firing of NREM sleep active vlPAG neurons contains information about future REM states that is not contained in the firing of REM sleep-active neurons. Based on these data, it may be more likely that NREM sleep active neurons are the recipients of REM sleep drive inputs. For most neurons participating in a flip-flop circuit arrangement, their activity will sharply accelerate/decelerate over the course of NRt bouts; however, some neurons receiving REM sleep drive inputs will exhibit gradual changes in firing rate over the span of the inter-REM interval. Directly recording from the later type of neuron is the most definitive means of determining sites of REM sleep drive integration. Weber et al. (2018) have reported that, within inter-REM intervals, the firing rate of GABAergic NREM sleep active neurons is higher for the first NREM bout in the interval relative to the last. Moreover, the firing rate of these neurons is elevated in the first NREM sleep bout following an episode of REM sleep in comparison to the NREM bout preceding the REM sleep episode, further evidence that the firing rate of GABAergic NREM sleep active neurons may relate to the accumulation/dissipation of REM sleep drive propensity. Critically, retrogradely tracing the pathways that input into sites of REM sleep drive integration may be a useful strategy toward identifying the physical substrate of REM sleep drive(s), mechanisms of REM sleep homeostasis and, potentially, REM sleep function.

The bulk of studies interrogating the neural circuitry of sleep adopt a reductionist approach, where the aim of any given experiment is to assign a well described functional role to a functionally distinct cell group. Focal cell inactivation using muscimol is regarded as inferior with respect to genetic targeting methods because the latter enable greater cellular specificity. Achieving cell type specificity is undeniably important; however, it is inappropriate to think that any method can be used to cleanly target individual functional groups. In other words, our a priori assumption should always be that experimental outcomes, at least potentially, reflect the modulation of multiple functional groups simultaneously. Therefore, focal inactivation using muscimol may be judged as a less appropriate tool, but only especially so where the results of which are used to attribute functional significance to a single cell group. However, this is not the aim of the current study. Here, we used simulated circuit dynamics and experimental manipulations thereof to detect contributions from multiple, functionally-related cell groups, namely the REM sleep-active and the REM sleep-inactive groups. Nevertheless, it should be noted that there are other cell groups in the in the ventral PAG region that contribute to sleep-wake regulation. For instance, the PAG contains a dopaminergic cell population that promotes wakefulness (Lu et al., 2006b) and a neurotensinergic subpopulation of glutamatergic cells that are not only active during NREM sleep, but may also promote the state through their innervation and excitation of a downstream population in caudal ventromedial medulla (Zhong et al., 2019). We observed a suppression of wakefulness during muscimol-mediated inhibition of the vlPAG/DpMe region, which could be accounted for by inhibition of the former dopaminergic cell group. Our finding that the distribution of NREM seep bout lengths was not markedly effected by vlPAG/DpMe inhibition is not consistent with modulation of the latter neurotensinergic cell group. Nevertheless, our use of pharmacological inhibition undoubtedly produced non-specific inhibition of multiple functional groups.

We cannot rule out that the possibility that the implantation of the microdialysis probes in our animals had an effect on baseline sleep dynamics as we did not include a control group of rats without implanted probes. Our experiments were conducted on singly housed animals; therefore, we cannot account for the impact of social isolation stress on our results. Data were collected between ZT3 and ZT7.5. Repeating this study at other times of day may produce different results.

Future studies are needed to target-specific cell groupings and more fully test the claims made by this study. For instance, in interpreting our findings, we assume that the effects we observed in NREM/REM sleep transitionary dynamics stem from underlying REM sleep-active and sleep-inactive GABAergic subpopulations of the vlPAG/DpMe. Therefore, we would predict that the results of our study would largely agree with those of a targeted inactivation of vlPAG/DpMe GABAergic neurons, where a similar analysis of NRt state dynamics is performed. The most critical test of our model predictions, and our claim that REM sleep-active and sleep-inactive subpopulations of the vlPAG/DpMe both contribute to REM sleep regulation through participation in a flip-flop switch, will be made by interventions that can successfully target neuronal groups on the basis of their firing rate activity profiles across the sleep-wake cycle [e.g., through further development of tools such as Capturing Activated Neural Ensembles (CANE) introduced by Sakurai et al., 2016]. Based on our study, specific inhibition (although not total inactivation) of GABAergic REM sleep-active neurons in the vlPAG/DpMe region would be expected to delay NREM-to-REM sleep transitioning and to disproportionately fragment REM sleep with bouts of NRt. In contrast, specific inhibition (although not total inactivation) of GABAergic REM sleep-inactive neurons in the vlPAG/DpMe region would be expected to reduce the threshold for transitioning between NREM and REM sleep while enhancing or preserving stability of the REM sleep state.

## Synthesis

Reviewing Editor: Liset Menendez de la Prida, Instituto Cajal CSIC Decisions are customarily a result of the Reviewing Editor and the peer reviewers coming together and discussing their recommendations until a consensus is reached. When revisions are invited, a fact-based synthesis statement explaining their decision and outlining what is needed to prepare a revision will be listed below. The following reviewer(s) agreed to reveal their identity: Franz Weber.

Your ms was reviewed by two independent referees and mysefl. We all value your careful combination of data analysis with modeling and agreed on the importance of your results. However, we have identified a number of points to be corrected. We feel you should be able to revise in less than two months and therefore we will be happpy to consider a major revsion.

Here is a list of concerns to be addressed:

1) Comments regarding definition of REM,NREM clusters and NRt. We feel definition and detection of NRt states is critical in your work. However, from the description in the methods it is hard to understand how exactly NRt and envelops are defined. We feel an additional figure illustrating the method and/or a more intuitive way of description could help to make is understandable to a more general audience. We also feel the algorithm could be made accesible.

2) Similarly, we found the computational approach very difficult to understand and ask you to expand this part in a different section. It would also be nice to clearly list all model predictions that may be validated experimentally in the future.

3) Muscimol injections affect both inhibitory and excitatory neurons and therefore effects may be unspecific. Recent work has highlighted the role of wake promoting and also NREM promoting subpopulation of glutamatergic neurons innervation GABAergic cells (which innervate GABAergic neurons; Zhong et al., 2019).It is thus unclear how pharmacological suppression of these subpopulations might or might not change their simulation results. Also, we feel the study would benefit from some true control experiment (no injection, just ephys).

4) Figures 1, 2 show only averages over two hour intervals. We wonder how much do the percentages of brain states change over time (within the two hour interval)? It would be very informative to examine time course (using finer time bins) of the brain state percentages.

5) We feel Figure 9 is very hard to intuitively understand. For example, data in panels A,B, and G are densily packed and number information is unclear. We feel more explanatory labels or symbols (e.g. indicating muscimol injection) might help. Please, clarify panel C also. In addition to help the read, it would great to show an example run of the model with the simulated firing rates of single neurons (or population) average.

6) How does NRt affect REM pressure in the model? We assume that REM pressure dissipates during REM states, but what happens during NRt and does this assumption in any way affect the results/conclusions?

7) we had concerns regarding singly housed animals. Instruction for authors indicate that you need to discuss the possible caveats. Social isolation has important effects on sleep patterns.

8) We strongly recommend using estimation statistics whenever possible in the paper. Bargraphs must be converted; all data points must be shown. Confidence intervals need to be provided.

9) We feel that the method section with the validation of the diffusion should be in the result section (perhaps you can start with it and the morphology).

10) Please edit the ms. There are many typos to correct.
