# Supplementary material for: A Focal Inactivation and Computational Study of Ventrolateral Periaqueductal Gray and Deep Mesencephalic Reticular Nucleus Involvement in Sleep State Switching and Bistability
Source: eNeuro. 2020 Nov 4;7(6):ENEURO.0451-19.2020. doi: 10.1523/ENEURO.0451-19.2020 (PMC7768273; doi:10.1523/ENEURO.0451-19.2020)
Supplement: Extended Data — Code accessibility statement. The included computer code is in four parts. First, is a MATLAB script entitled “flip_flop_circuit_simulation_initializer.” This code was used to initialize simulations run with SimLIFnet (available for download at https://www.mathworks.com/matlabcentral/fileexchange/50339; copyright 2015, Zachary Danziger, all rights reserved) using the simulation parameters listed in Extended Data Table 8-1. Second is a MATLAB function entitled “forceramp,” which is required by “flip_flop_circuit_simulation_initializer” and determines the profile of the R-state promoting drive. Third, is a MATLAB script entitled “intersection_finder,” which was used to identifying all points in NREM/REM state space that bound trajectory intersections occurring within 1-min-wide windows. This procedure is needed to demarcate NREM, REM, and NRt regions of state space. Fourth, is a MATLAB script entitled “drug_diffusion_simulations,” which was used to estimate the 3-dimenional spread of drug from a point source in a microinjection versus a reverse-microdialysis scenario. This code is freely available online at https://github.com/KPGrace/Grace_Horner_Eneuro2020. Download Extended Data, ZIP file. [file enu-eN-NWR-0451-19-s05.zip › Drug Diffusion Simulations.docx]

%Drug Diffusion Simulations: Microinjection vs. Microdialysis

clear all

t=150; %total time in 'minutes'

mit=30; %microinjection time in 'minutes' (set to 30)

miu=307; %microinjection [base]

mdu=0.085; %microdialysis [base]

ks=3; %Gaussian kernel size

sd=1.2; %Gaussian standard deviation

mispace(250,250,250)=0; %microinjection volume

mdspace(250,250,250)=0; %microdialysis volume

%set initial 'seed' concentrations in the central cell

mispace(125,125,125)=miu;

mdspace(125,125,125)=mdu;

%%

%simulation

for i=1:mit;

mispace = smooth3(mispace,'gaussian',ks,sd);

mdspace = smooth3(mdspace,'gaussian',ks,sd);

ini=mispace(125,125,125); %get seed concentration after smoothing

mispace(125,125,125)=ini+miu;

mdspace(125,125,125)=mdu;

end

% after the first 30 iterations, concentration in the mispace is maximum

% and will now begin to decline (i.e.,we stop adding to the microinjection

% seed volume. Meanwhile, concentrations in the mdspace will continue to rise.

for i=1:t-mit;

mispace = smooth3(mispace,'gaussian',ks,sd);

mdspace = smooth3(mdspace,'gaussian',ks,sd);

mdspace(125,125,125)=mdu;

end

dslice=mdspace(:,:,125); %get central 2-d plane from microdialysis simulation

islice=mispace(:,:,125); %get central 2-d plane from microinjection simulation

dslice_percent=dslice*1000000; %convert to nM

islice_percent=islice*1000000; %convert to nM

dslice_percent=(4*exp(-7)).*(dslice_percent.^3)-(0.0007.*(dslice_percent.^2))+(0.45.*dslice_percent); %convert to %spike inhibition

islice_percent=(4*exp(-7)).*(islice_percent.^3)-(0.0007.*(islice_percent.^2))+(0.45.*islice_percent);

dslice_percent(dslice_percent<1)=0;

dslice_percent(dslice_percent>100)=100;

islice_percent(islice_percent<1)=0;

islice_percent(islice_percent>100)=100;
