# Supplementary material for: A Focal Inactivation and Computational Study of Ventrolateral Periaqueductal Gray and Deep Mesencephalic Reticular Nucleus Involvement in Sleep State Switching and Bistability
Source: eNeuro. 2020 Nov 4;7(6):ENEURO.0451-19.2020. doi: 10.1523/ENEURO.0451-19.2020 (PMC7768273; doi:10.1523/ENEURO.0451-19.2020)
Supplement: Extended Data — Code accessibility statement. The included computer code is in four parts. First, is a MATLAB script entitled “flip_flop_circuit_simulation_initializer.” This code was used to initialize simulations run with SimLIFnet (available for download at https://www.mathworks.com/matlabcentral/fileexchange/50339; copyright 2015, Zachary Danziger, all rights reserved) using the simulation parameters listed in Extended Data Table 8-1. Second is a MATLAB function entitled “forceramp,” which is required by “flip_flop_circuit_simulation_initializer” and determines the profile of the R-state promoting drive. Third, is a MATLAB script entitled “intersection_finder,” which was used to identifying all points in NREM/REM state space that bound trajectory intersections occurring within 1-min-wide windows. This procedure is needed to demarcate NREM, REM, and NRt regions of state space. Fourth, is a MATLAB script entitled “drug_diffusion_simulations,” which was used to estimate the 3-dimenional spread of drug from a point source in a microinjection versus a reverse-microdialysis scenario. This code is freely available online at https://github.com/KPGrace/Grace_Horner_Eneuro2020. Download Extended Data, ZIP file. [file enu-eN-NWR-0451-19-s05.zip › flip_flop_circuit_simulation_initializer.docx]

%flip_flop_circuit_simulation_initializer script

%To be used for initializing Flip-flop switch simulations using SimLIFnet,

%used for the design and simulation of leaky integrate and fire (LIF)

%neuron networks. SimLIFnet available for download at:

%https://www.mathworks.com/matlabcentral/fileexchange/50339 (Copyright © 2015, Zachary Danziger, All rights reserved).

% OUTPUT:

%(Binned_spikes) data structure containing the average population firing rates

% of the N and R pools for every network and every experiment

%(NetParams) data structure containing the simulation parameters

%Defaults

nets=60; %total number of networks that will be generated

p=0.5; %flip-flop switch connection probability

noise=1.75; %flip-flop neuron current noise

synaptic_density=4; % same as SimLIFnet default value

total_t=400; %simulation time in 'seconds'

bin_no=400; %number of bins for spike averaging

Ts=0.05; %time step per simulation iteration

% Offset Current Values for R and N pools respectively

% Increase or decreasing baseline values will have major effects on initial

% flip-flop behavior. Increasing or decreasing values from baseline represent neuronal activation and inactivation respectively.

base=[2 2]; % sets 1 & 5

inhibit_R=[1.85 2]; % sets 2 & 6

inhibit_N=[2 1.85]; % sets 3 & 7

inhibit_N_R=[1.85 1.85]; % sets 4 & 8

for k=1:nets;

figure

%%%%%%%%%%%%%%%%%%%%%%%%%%%%%%%%%%%%%%

%%%%%%%%%%%%%%%%%%%%%%%%%%%%%%%%%%%%%%

% create network connectivity matrix %

%%%%%%%%%%%%%%%%%%%%%%%%%%%%%%%%%%%%%%

%%%%%%%%%%%%%%%%%%%%%%%%%%%%%%%%%%%%%%

%%create the FLIP-FLOP block (size=50x50, 2 groups of 25 neurons)

W2=rand(50,50);

W2(W2<p)=0;

W2=round(W2);

%%Set random weights

R=rand(50,50);

W2=W2.*R;

W2=W2.*-1; %make connections inhibitory

clear R

W2(1:25,1:25)=0; %remove all R-pool auto-inhibition

W2(26:50,26:50)=0; %remove all N-pool auto-inhibition

W2(1:25,26:50)=W2(1:25,26:50)./2.5;% downscale R-->N Note that changing these scaling values will strongly influence flip-flop behavior

W2(26:50,1:25)=W2(26:50,1:25)./2.2;% downscale N-->R

%%create the INput block of the connectivity matrix

%%make connections inhibitory for simulation sets 1-4 (ramping input delivered through N-pool)

%downscale initial weights

W3=((ones(5,25)).*-1)./60;

%Combine the blocks above into a single connectivity matrix

%(columns correspond to R?N weighting)(rows correspond to N?R weighting)

W(60,60)=0; % create blank array for whole network (2x5input,25xR,25xN)

W(11:60,11:60)=W2; %insert flip-flop

W(1:5,36:60)=W3; %insert INramp to flip-OFF weights

W(6:10,11:35)=W3; %insert INno-ramp to flip-ON weights

IC(60,1)=0; %create blank array for initial conditions

IC(36:60,1)=100; %set initial voltages for the N-pool neurons (initiates the switch in the N-state)

off(60,1)=0; %create blank array for offset currents

off(1:5,1)=2.125; %set offsets currents for inputs to N-pool

off(6:10,1)=2.125; %set offsets currents for inputs to R-pool

n(60,1)=0; %create blank array for noise amplitudes

n(1:10,1)=0; %set noise for input neurons

n(11:60,1)=noise; %set noise for flip-flop groups

synd(1:60,1:60)=synaptic_density; %set synaptic density (SimLIFnet default =4)

%%

%%%%%%%%%%%%%%%%%%%%%%%%%%%%%%%%%%%%%%

%%%%%%%%%%%%%%%%%%%%%%%%%%%%%%%%%%%%%%

% BASELINE (1) %

%%%%%%%%%%%%%%%%%%%%%%%%%%%%%%%%%%%%%%

%%%%%%%%%%%%%%%%%%%%%%%%%%%%%%%%%%%%%%

NETWORK=k

SET=1

off(11:35,1)=base(1); %set offset currents for flip-flop R-pool

off(36:60,1)=base(2); %set offset currents for flip-flop N-pool

%initialize R-state drive input through N-pool inputs (neurons 1-5)

F = {@(t) forceramp(t),1; ...

@(t) forceramp(t),2; ...

@(t) forceramp(t),3; ...

@(t) forceramp(t),4; ...

@(t) forceramp(t),5};

%RUN the simulation

[spk NetParams V] = SimLIFNet(W,'simTime',total_t,'offsetCurrents',off,...

'forcingFunctions',F,'noiseAmplitude',n,'tstep',Ts,'initialConditions',IC,...

'plotResults',0,'synapticDensity',synd);

%Bin the spikes

bins=0:1:bin_no;

for i=1:50;

spks=spk{i+10,1};

[h,binsout] = hist(spks,bins);

hall(i,:)=h;

end

%Calculate the average population firing rate in the R and N pools

havg_Rpool = mean(hall(1:25,:));

havg_Npool = mean(hall(26:50,:));

%plot the results

subplot(2,4,1);

hold on;

plot(havg_Rpool,'DisplayName','Rpool');

plot(havg_Npool,'DisplayName','Npool');

%Store the average population firing rates in a data structure

Binned_spikes.input_to_N.Rpool.baseline(k,:,1)=havg_Rpool;

Binned_spikes.input_to_N.Npool.baseline(k,:,1)=havg_Npool;

%%

%%%%%%%%%%%%%%%%%%%%%%%%%%%%%%%%%%%%%%%%%%%

%%%%%%%%%%%%%%%% set 2 %%%%%%%%%%%%%%%%%%%%

%%%%%%%%%%%%%%%%%%%%%%%%%%%%%%%%%%%%%%%%%%%

clear spks h hall

NETWORK=k

SET=2

off(11:35,1)=inhibit_R(1); %set offset currents for flip-flop R-pool

off(36:60,1)=inhibit_R(2); %set offset currents for flip-flop N-pool

[spk NetParams V] = SimLIFNet(W,'simTime',400,'offsetCurrents',off,...

'forcingFunctions',F,'noiseAmplitude',n,'tstep',0.05,'initialConditions',IC,...

'plotResults',0,'synapticDensity',synd);

%Bin the spikes

bins=0:1:bin_no;

for i=1:50;

spks=spk{i+10,1};

[h,binsout] = hist(spks,bins);

hall(i,:)=h;

end

%Calculate the average population firing rate in the R and N pools

havg_Rpool = mean(hall(1:25,:));

havg_Npool = mean(hall(26:50,:));

%plot the results

subplot(2,4,2);

hold on;

plot(havg_Rpool,'DisplayName','Rpool');

plot(havg_Npool,'DisplayName','Npool');

%Store the average population firing rates in a data structure

Binned_spikes.input_to_N.Rpool.set2(k,:,1)=havg_Rpool;

Binned_spikes.input_to_N.Npool.set2(k,:,1)=havg_Npool;

%%

%%%%%%%%%%%%%%%%%%%%%%%%%%%%%%%%%%%%%%%%%%%

%%%%%%%%%%%%%%%% set 3 %%%%%%%%%%%%%%%%%%%%

%%%%%%%%%%%%%%%%%%%%%%%%%%%%%%%%%%%%%%%%%%%

clear spks h hall

NETWORK=k

SET=3

off(11:35,1)=inhibit_N(1); %set offset currents for flip-flop R-pool

off(36:60,1)=inhibit_N(2); %set offset currents for flip-flop N-pool

[spk NetParams V] = SimLIFNet(W,'simTime',400,'offsetCurrents',off,...

'forcingFunctions',F,'noiseAmplitude',n,'tstep',0.05,'initialConditions',IC,...

'plotResults',0,'synapticDensity',synd);

%Bin the spikes

bins=0:1:bin_no;

for i=1:50;

spks=spk{i+10,1};

[h,binsout] = hist(spks,bins);

hall(i,:)=h;

end

%Calculate the average population firing rate in the R and N pools

havg_Rpool = mean(hall(1:25,:));

havg_Npool = mean(hall(26:50,:));

%plot the results

subplot(2,4,3);

hold on;

plot(havg_Rpool,'DisplayName','Rpool');

plot(havg_Npool,'DisplayName','Npool');

%Store the average population firing rates in a data structure

Binned_spikes.input_to_N.Rpool.set3(k,:,1)=havg_Rpool;

Binned_spikes.input_to_N.Npool.set3(k,:,1)=havg_Npool;

%%

%%%%%%%%%%%%%%%%%%%%%%%%%%%%%%%%%%%%%%%%%%%

%%%%%%%%%%%%%%%% set 4 %%%%%%%%%%%%%%%%%%%%

%%%%%%%%%%%%%%%%%%%%%%%%%%%%%%%%%%%%%%%%%%%

clear spks h hall

NETWORK=k

SET=4

off(11:35,1)=inhibit_N_R(1); %set offset currents for flip-flop R-pool

off(36:60,1)=inhibit_N_R(2); %set offset currents for flip-flop N-pool

[spk NetParams V] = SimLIFNet(W,'simTime',400,'offsetCurrents',off,...

'forcingFunctions',F,'noiseAmplitude',n,'tstep',0.05,'initialConditions',IC,...

'plotResults',0,'synapticDensity',synd);

%Bin the spikes

bins=0:1:bin_no;

for i=1:50;

spks=spk{i+10,1};

[h,binsout] = hist(spks,bins);

hall(i,:)=h;

end

%Calculate the average population firing rate in the R and N pools

havg_Rpool = mean(hall(1:25,:));

havg_Npool = mean(hall(26:50,:));

%plot the results

subplot(2,4,4);

hold on;

plot(havg_Rpool,'DisplayName','Rpool');

plot(havg_Npool,'DisplayName','Npool');

%Store the average population firing rates in a data structure

Binned_spikes.input_to_N.Rpool.set4(k,:,1)=havg_Rpool;

Binned_spikes.input_to_N.Npool.set4(k,:,1)=havg_Npool;

%%

%%%%%%%%%%%%%%%%%%%%%%%%%%%%%%%%%%%%%%

%%%%%%%%%%%%%%%%%%%%%%%%%%%%%%%%%%%%%%

% BASELINE (5) %

%%%%%%%%%%%%%%%%%%%%%%%%%%%%%%%%%%%%%%

%%%%%%%%%%%%%%%%%%%%%%%%%%%%%%%%%%%%%%

clear spks h hall

NETWORK=k

SET=5

%for simulation sets 5-8, the R-state drive is delivered through the

%R-pool, which requires that the input neurons be excitatory

W3=W3.*-1; %make input connections excitatory (W3 contains negative values initially)

W(1:5,36:60)=W3; %insert INramp to flip-OFF weights

W(6:10,11:35)=W3; %insert INno-ramp to flip-ON weights

off(11:35,1)=base(1); %set offset currents for flip-flop R-pool

off(36:60,1)=base(2); %set offset currents for flip-flop N-pool

F = {@(t) forceramp(t),6; ...

@(t) forceramp(t),7; ...

@(t) forceramp(t),8; ...

@(t) forceramp(t),9; ...

@(t) forceramp(t),10};

[spk NetParams V] = SimLIFNet(W,'simTime',400,'offsetCurrents',off,...

'forcingFunctions',F,'noiseAmplitude',n,'tstep',0.05,'initialConditions',IC,...

'plotResults',0,'synapticDensity',synd);

%Bin the spikes

bins=0:1:bin_no;

for i=1:50;

spks=spk{i+10,1};

[h,binsout] = hist(spks,bins);

hall(i,:)=h;

end

%Calculate the average population firing rate in the R and N pools

havg_Rpool = mean(hall(1:25,:));

havg_Npool = mean(hall(26:50,:));

%plot the results

subplot(2,4,5);

hold on;

plot(havg_Rpool,'DisplayName','Rpool');

plot(havg_Npool,'DisplayName','Npool');

%Store the average population firing rates in a data structure

Binned_spikes.input_to_R.Rpool.baseline5(k,:,1)=havg_Rpool;

Binned_spikes.input_to_R.Npool.baseline5(k,:,1)=havg_Npool;

%%

%%%%%%%%%%%%%%%%%%%%%%%%%%%%%%%%%%%%%%%%%%%

%%%%%%%%%%%%%%%% set 6 %%%%%%%%%%%%%%%%%%%%

%%%%%%%%%%%%%%%%%%%%%%%%%%%%%%%%%%%%%%%%%%%

clear spks h hall

NETWORK=k

SET=6

off(11:35,1)=inhibit_R(1); %set offset currents for flip-flop R-pool

off(36:60,1)=inhibit_R(2); %set offset currents for flip-flop N-pool

[spk NetParams V] = SimLIFNet(W,'simTime',400,'offsetCurrents',off,...

'forcingFunctions',F,'noiseAmplitude',n,'tstep',0.05,'initialConditions',IC,...

'plotResults',0,'synapticDensity',synd);

%Bin the spikes

bins=0:1:bin_no;

for i=1:50;

spks=spk{i+10,1};

[h,binsout] = hist(spks,bins);

hall(i,:)=h;

end

%Calculate the average population firing rate in the R and N pools

havg_Rpool = mean(hall(1:25,:));

havg_Npool = mean(hall(26:50,:));

%plot the results

subplot(2,4,6);

hold on;

plot(havg_Rpool,'DisplayName','Rpool');

plot(havg_Npool,'DisplayName','Npool');

%Store the average population firing rates in a data structure

Binned_spikes.input_to_R.Rpool.set6(k,:,1)=havg_Rpool;

Binned_spikes.input_to_R.Npool.set6(k,:,1)=havg_Npool;

%%

%%%%%%%%%%%%%%%%%%%%%%%%%%%%%%%%%%%%%%%%%%%

%%%%%%%%%%%%%%%% set 7 %%%%%%%%%%%%%%%%%%%%

%%%%%%%%%%%%%%%%%%%%%%%%%%%%%%%%%%%%%%%%%%%

clear spks h hall

NETWORK=k

SET=7

off(11:35,1)=inhibit_N(1); %set offset currents for flip-flop R-pool

off(36:60,1)=inhibit_N(2); %set offset currents for flip-flop N-pool

[spk NetParams V] = SimLIFNet(W,'simTime',400,'offsetCurrents',off,...

'forcingFunctions',F,'noiseAmplitude',n,'tstep',0.05,'initialConditions',IC,...

'plotResults',0,'synapticDensity',synd);

%Bin the spikes

bins=0:1:bin_no;

for i=1:50;

spks=spk{i+10,1};

[h,binsout] = hist(spks,bins);

hall(i,:)=h;

end

%Calculate the average population firing rate in the R and N pools

havg_Rpool = mean(hall(1:25,:));

havg_Npool = mean(hall(26:50,:));

%plot the results

subplot(2,4,7);

hold on;

plot(havg_Rpool,'DisplayName','Rpool');

plot(havg_Npool,'DisplayName','Npool');

%Store the average population firing rates in a data structure

Binned_spikes.input_to_R.Rpool.set7(k,:,1)=havg_Rpool;

Binned_spikes.input_to_R.Npool.set7(k,:,1)=havg_Npool;

%%

%%%%%%%%%%%%%%%%%%%%%%%%%%%%%%%%%%%%%%%%%%%

%%%%%%%%%%%%%%%% set 8 %%%%%%%%%%%%%%%%%%%%

%%%%%%%%%%%%%%%%%%%%%%%%%%%%%%%%%%%%%%%%%%%

clear spks h hall

NETWORK=k

SET=8

off(11:35,1)=inhibit_N_R(1); %set offset currents for flip-flop R-pool

off(36:60,1)=inhibit_N_R(2); %set offset currents for flip-flop N-pool

[spk NetParams V] = SimLIFNet(W,'simTime',400,'offsetCurrents',off,...

'forcingFunctions',F,'noiseAmplitude',n,'tstep',0.05,'initialConditions',IC,...

'plotResults',0,'synapticDensity',synd);

%Bin the spikes

bins=0:1:bin_no;

for i=1:50;

spks=spk{i+10,1};

[h,binsout] = hist(spks,bins);

hall(i,:)=h;

end

%Calculate the average population firing rate in the R and N pools

havg_Rpool = mean(hall(1:25,:));

havg_Npool = mean(hall(26:50,:));

%plot the results

subplot(2,4,8);

hold on;

plot(havg_Rpool,'DisplayName','Rpool');

plot(havg_Npool,'DisplayName','Npool');

%Store the average population firing rates in a data structure

Binned_spikes.input_to_R.Rpool.set8(k,:,1)=havg_Rpool;

Binned_spikes.input_to_R.Npool.set8(k,:,1)=havg_Npool;

end

clearvars -except NetParams Binned_spikes
