# Supplementary material for: A Focal Inactivation and Computational Study of Ventrolateral Periaqueductal Gray and Deep Mesencephalic Reticular Nucleus Involvement in Sleep State Switching and Bistability
Source: eNeuro. 2020 Nov 4;7(6):ENEURO.0451-19.2020. doi: 10.1523/ENEURO.0451-19.2020 (PMC7768273; doi:10.1523/ENEURO.0451-19.2020)
Supplement: Extended Data — Code accessibility statement. The included computer code is in four parts. First, is a MATLAB script entitled “flip_flop_circuit_simulation_initializer.” This code was used to initialize simulations run with SimLIFnet (available for download at https://www.mathworks.com/matlabcentral/fileexchange/50339; copyright 2015, Zachary Danziger, all rights reserved) using the simulation parameters listed in Extended Data Table 8-1. Second is a MATLAB function entitled “forceramp,” which is required by “flip_flop_circuit_simulation_initializer” and determines the profile of the R-state promoting drive. Third, is a MATLAB script entitled “intersection_finder,” which was used to identifying all points in NREM/REM state space that bound trajectory intersections occurring within 1-min-wide windows. This procedure is needed to demarcate NREM, REM, and NRt regions of state space. Fourth, is a MATLAB script entitled “drug_diffusion_simulations,” which was used to estimate the 3-dimenional spread of drug from a point source in a microinjection versus a reverse-microdialysis scenario. This code is freely available online at https://github.com/KPGrace/Grace_Horner_Eneuro2020. Download Extended Data, ZIP file. [file enu-eN-NWR-0451-19-s05.zip › forceramp.docx]

%R-state drive input:

%to be used in conjunction with the

%Flip-flop switch simulation initializer script

function f = forceramp(t)

if t<100 ;

f=0;

else

f = (t-100)*0.06;

end
