# Supplementary material for: A Focal Inactivation and Computational Study of Ventrolateral Periaqueductal Gray and Deep Mesencephalic Reticular Nucleus Involvement in Sleep State Switching and Bistability
Source: eNeuro. 2020 Nov 4;7(6):ENEURO.0451-19.2020. doi: 10.1523/ENEURO.0451-19.2020 (PMC7768273; doi:10.1523/ENEURO.0451-19.2020)
Supplement: Extended Data — Code accessibility statement. The included computer code is in four parts. First, is a MATLAB script entitled “flip_flop_circuit_simulation_initializer.” This code was used to initialize simulations run with SimLIFnet (available for download at https://www.mathworks.com/matlabcentral/fileexchange/50339; copyright 2015, Zachary Danziger, all rights reserved) using the simulation parameters listed in Extended Data Table 8-1. Second is a MATLAB function entitled “forceramp,” which is required by “flip_flop_circuit_simulation_initializer” and determines the profile of the R-state promoting drive. Third, is a MATLAB script entitled “intersection_finder,” which was used to identifying all points in NREM/REM state space that bound trajectory intersections occurring within 1-min-wide windows. This procedure is needed to demarcate NREM, REM, and NRt regions of state space. Fourth, is a MATLAB script entitled “drug_diffusion_simulations,” which was used to estimate the 3-dimenional spread of drug from a point source in a microinjection versus a reverse-microdialysis scenario. This code is freely available online at https://github.com/KPGrace/Grace_Horner_Eneuro2020. Download Extended Data, ZIP file. [file enu-eN-NWR-0451-19-s05.zip › Intersection Finder.docx]

%Intersection Finder:

%Identifying all points in state-space that bound trajectory intersections occuring within one minute windows

%Required Arrays to start:

%%%%%% 1)array entitled 'xs': x-axis coordinate values (arranged row-wise)

%%%%%% 2)array entitled 'ys': y-axis coordinate values

%Output:

% new versions of arrays 'xs' and 'xy', where coordinate points not bounding

% intersections are converted to zeros

s=size(xs,1)-15; % get size of data set

surrounding_points=zeros(size(xs,1),1); % preallocate space

for i=1:s

if xs(i)==0 || xs(i+1)==0;

continue

end

for n=2:5 %look for trajectory intersections between the 'current epoch line' and the future epoch lines for the next minute

if xs(i+n)==0 || xs(i+n+1)==0;

continue

end

%% set the coordinates of the current and future epoch lines

line1=[xs(i) ys(i); xs(i+1) ys(i+1)];

line2=[xs(i+n) ys(i+n); xs(i+n+1) ys(i+n+1)];

% calculate slope for current and future epoch lines

slope = @(line) (line(2,2) - line(1,2))/(line(2,1) - line(1,1));

m1 = slope(line1);

m2 = slope(line2);

% Determine the coordinates where the lines intersect

intercept = @(line,m) line(1,2) - m*line(1,1);

b1 = intercept(line1,m1);

b2 = intercept(line2,m2);

xintersect = (b2-b1)/(m1-m2);

yintersect = m1*xintersect + b1;

% Determine if the point of intersection is, or is not, bounded by the data points

isPointInside = @(xint,myline) ...

(xint >= myline(1,1) && xint <= myline(2,1)) || ...

(xint >= myline(2,1) && xint <= myline(1,1));

inside = isPointInside(xintersect,line1) && ...

isPointInside(xintersect,line2);

%keep track of the bounded intersections

if inside==1

Xintersection(i,n)=xintersect;

Yintersection(i,n)=yintersect;

surrounding_points(i,1)=1;

surrounding_points(i+1,1)=1;

surrounding_points(i+n,1)=1;

surrounding_points(i+n+1,1)=1;

end

end

end

XXintersection=transpose(Xintersection);

YYintersection=transpose(Yintersection);

XXXintersection=reshape(XXintersection,[],1);

YYYintersection=reshape(YYintersection,[],1);

% index the data point coordinates that bound trajectory intersections

xs=surrounding_points.*xs;

ys=surrounding_points.*ys;

clearvars -except xs xy
