# Supplementary figures and images for: A Focal Inactivation and Computational Study of Ventrolateral Periaqueductal Gray and Deep Mesencephalic Reticular Nucleus Involvement in Sleep State Switching and Bistability
Source: eNeuro. 2020 Nov 4;7(6):ENEURO.0451-19.2020. doi: 10.1523/ENEURO.0451-19.2020 (PMC7768273; doi:10.1523/ENEURO.0451-19.2020)

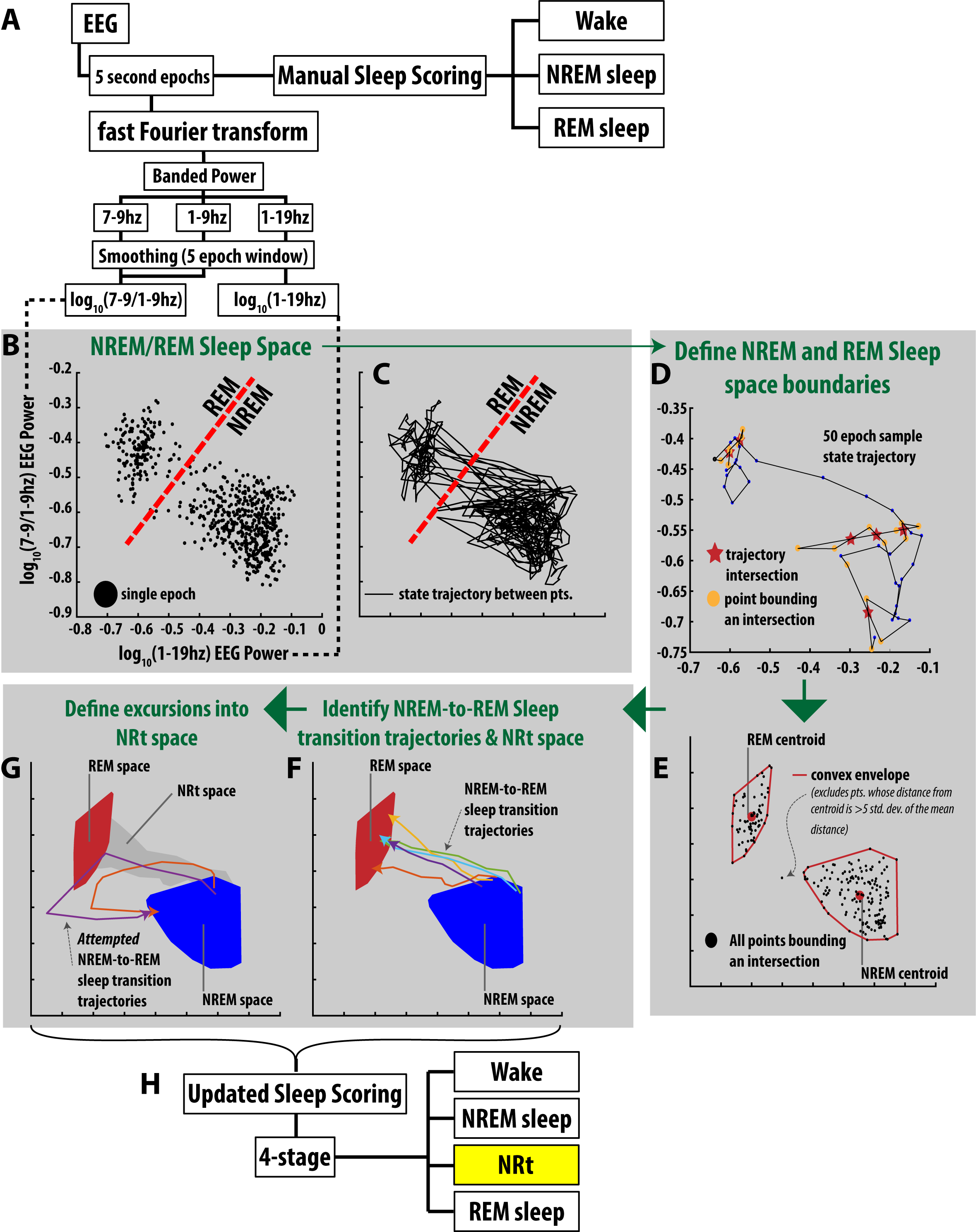

Supplement: Extended Data Figure 2-1 — Procedure for defining NREM-to-REM sleep transition dynamics. A, Flow chart outlining the EEG preprocessing steps required to construct NREM/REM state-space plots. B, C, Example state-space plots showing clusters of points corresponding to REM and NREM sleep. D, E, Procedure for defining the boundaries of the REM and NREM clusters that separate them from the intervening NRt space. This procedure identifies the position of trajectory intersections within 5-epoch spans. Example trajectory intersections are shown in D. All data points that bound all such trajectory intersections are fitted with a convex envelope to form the REM sleep and NREM sleep boundaries shown in E. F, G, Example trajectories of complete NREM-to-REM transitions (F) and the trajectories of failed transitions through NRt space (G). Download Figure 2-1, TIF file. [file enu-eN-NWR-0451-19-s02.tif]

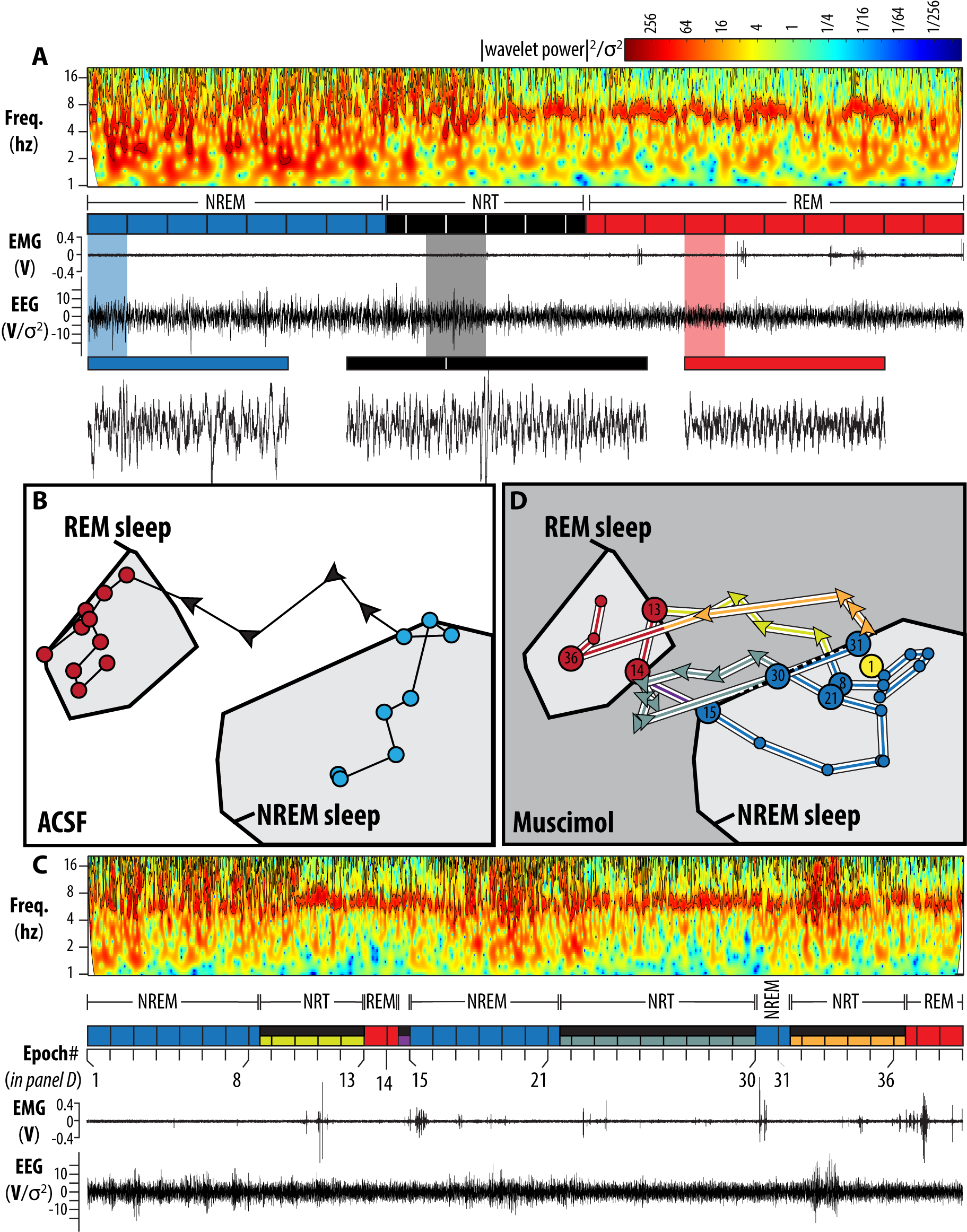

Supplement: Extended Data Figure 7-1 — Examples of normal and unstable EEG dynamics across transitions between NREM and REM sleep. A, Example spectrogram and corresponding state-space plot depicting normal spectral changes in the EEG across a NREM-to-REM sleep transition during ACSF microperfusion of the vlPAG/DpMe. B, The corresponding state-space plot for the EEG and EMG traces shown in A. The state-space trajectory moves: (i) from NREM sleep (one blue dot per epoch within NREM sleep boundary), (ii) into transitionary space (one black arrow per epoch; arrow direction indicating the direction of the state-space trajectory), (iii) and finishes within the REM sleep boundary. C, Example spectrogram and corresponding state-space plot depicting a period of unstable sleep during muscimol-mediated inhibition of the vlPAG/DpMe. D, The corresponding state-space plot for the EEG and EMG traces shown in C. The colors for the state trajectory segments correspond to the hypnogram colors in C. The trajectory begins in NREM sleep at point 1, moves into transitionary space during epoch 8, moves into REM sleep space at epoch 13, completes a transition from REM to NREM sleep space at epoch 15, moves back into transitionary space during epoch 21, re-enters NREM sleep space at epoch 30 having failed to enter REM sleep space, remains in NREM until epoch 31 before re-entering transition space and completing a transition into REM sleep space at epoch 36. Download Figure 7-1, TIF file. [file enu-eN-NWR-0451-19-s03.tif]

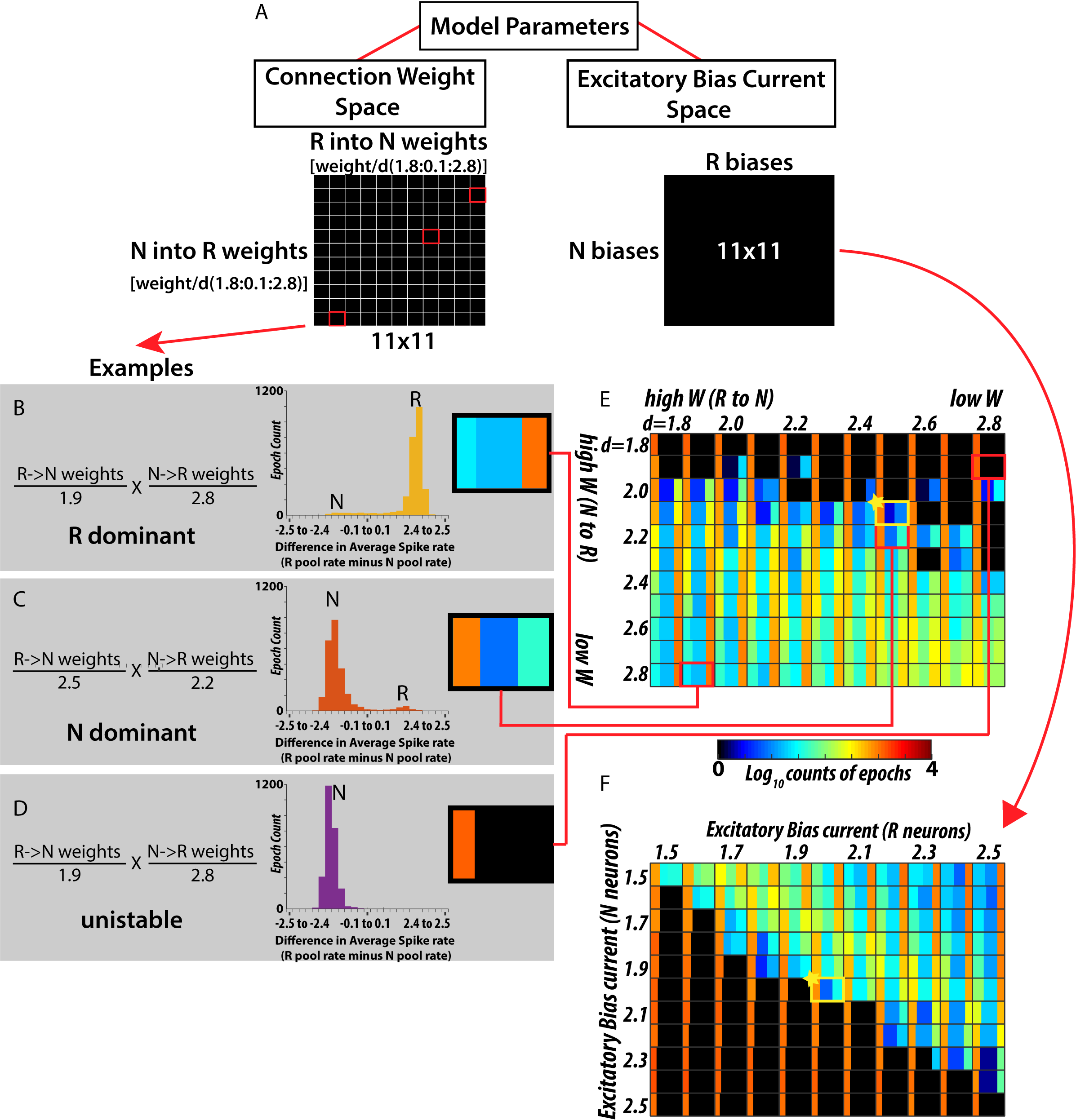

Supplement: Extended Data Figure 8-2 — Computer Simulations of flip-flop circuits: setting flip-flop synaptic weights and excitatory bias currents. A, Connection weighting was tuned prior to setting the level of excitatory bias current. Initial connection weights were divided by a factor, d, ranging from 1.8 to 2.8 in 0.1-unit increments. Nj→Ri weights were changed independent of Rj→Ni weights. A total of 121 combinations of Nj→Ri of and Rj→Ni weighting were used (Extended Data Fig. 9-1A). B–D, Examples (3/121) of the simulated combinations of flip-flop weighting. For each weighting combination, we calculated the difference in population firing rate over time and converted these data to a histogram showing the frequencies of binned R-N firing rate differences. Bimodal histograms indicate flip-flops that spontaneously switch between N-state and R-state. The height of the left and right peaks of the histograms indicate the prevalence of the N-state and R-state, respectively. The height of the intervening trough indicates the prevalence of N/R intermediate states. E, F, 11 × 11 simulation spaces where individual cells in the grid correspond to a given parameter combination and the color coding is a representation of the firing rate histogram generated from that particular set of simulations: the height of the N-peak, N/R trough, and the R-peak are indicated by the color of the left, middle, and right bars, respectively. The parameter combinations selected for experimental simulations are outlined in yellow. E, The stimulation space for synaptic weight (columns correspond to R→N weighting; rows correspond to N→R weighting). F, The simulation space for excitatory bias current (columns correspond to R neuron current; columns correspond to N neuron current). Download Figure 8-2, TIF file. [file enu-eN-NWR-0451-19-s04.tif]
