# Supplementary material for: A Focal Inactivation and Computational Study of Ventrolateral Periaqueductal Gray and Deep Mesencephalic Reticular Nucleus Involvement in Sleep State Switching and Bistability
Source: eNeuro. 2020 Nov 4;7(6):ENEURO.0451-19.2020. doi: 10.1523/ENEURO.0451-19.2020 (PMC7768273; doi:10.1523/ENEURO.0451-19.2020)
Supplement: Extended Data Table 8-1 — Parameters used for computer simulations of flip-flop circuits. A listing of all the parameters used to produce the flip-flop circuit simulations. Download Table 8-1, DOCX file. [file enu-eN-NWR-0451-19-s01.docx]

| Table 8-1 | | | | | | | | |
| --- | --- | --- | --- | --- | --- | --- | --- | --- |
|  | Experiment # (from Figure 8) | | | | | | | |
|  | 1 | 2 | 3 | 4 | 5 | 6 | 7 | 8 |
| Iapplied: ramping current | +0.06/iteration | | | | | | | |
| Iapplied: Excitatory bias current (N-pool) | 2 | 2 | 1.85 | 1.85 | 2 | 2 | 1.85 | 1.85 |
| Iapplied: Excitatory bias current (R-pool) | 2 | 1.85 | 2 | 1.85 | 2 | 1.85 | 2 | 1.85 |
| Iapplied: Noise amplitude | 1.5 | | | | | | | |
| Size of flip-flop pools | 25/pool | | | | | | | |
| Flip-flop pool connection probability | 0.5 | | | | | | | |
| N-pool to R-pool connection weight range | 0 to -0.48 (*d* = 2.1), | | | | | | | |
| R-pool to N-pool connection weight range | 0 to -0.4 (*d* = 2.5)) | | | | | | | |
| N-pool to N-pool connection weight range | 0 | | | | | | | |
| R-pool to R-pool connection weight range | 0 | | | | | | | |
| Input neuron to R-pool connection weight | 0 | | | | 1/60 | | | |
| Input neuron to N-pool connection weight | 1/60 | | | | 0 | | | |
| Firing rate difference threshold (R-pool firing – N-pool) for scoring the N-state | <-0.67 | | | | <-0.76 | | | |
| Firing rate difference threshold (R-pool firing – N-pool) for scoring the R-state | >0.907 | | | | >1.467 | | | |
| tstep : Simulated time step-size | 0.05 | | | | | | | |
| The amount of simulated time over which to integrate the model, in units of membrane time constants | 400 | | | | | | | |
| synapticDensity | 4 (SimLIFNet default) | | | | | | | |

**Table 8-1 Parameters used for computer simulations of flip-flop circuits**

A listing of all the parameters used to produce the flip-flop circuit simulations.
